# Assessment of performance characteristics of COVID-19 ICD-10-CM diagnosis code U07.1 using SARS-CoV-2 nucleic acid amplification test results

**DOI:** 10.1371/journal.pone.0273196

**Published:** 2022-08-18

**Authors:** Keran Moll, Shayan Hobbi, Cindy Ke Zhou, Kathryn Fingar, Timothy Burrell, Veronica Hernandez-Medina, Joyce Obidi, Nader Alawar, Steven A. Anderson, Hui-Lee Wong, Azadeh Shoaibi

**Affiliations:** 1 IBM Consulting, Bethesda, Maryland, United States of America; 2 U.S. Food and Drug Administration, Center for Biologics Evaluation and Research, Silver Spring, Maryland, United States of America; Universita degli Studi di Parma, ITALY

## Abstract

The Food and Drug Administration’s Biologics Effectiveness and Safety Initiative conducts active surveillance to protect public health during the coronavirus disease 2019 (COVID-19) pandemic. This study evaluated performance of International Classification of Diseases, Tenth Revision, Clinical Modification (ICD-10-CM) diagnosis code U07.1 in identifying COVID-19 cases in claims compared with severe acute respiratory syndrome coronavirus 2 (SARS-CoV-2) nucleic acid amplification test results in linked electronic health records (EHRs). Care episodes in three populations were defined using COVID-19-related diagnoses (population 1), SARS-CoV-2 nucleic acid amplification test procedures (population 2), and all-cause hospitalizations (population 3) in two linked claims-EHR databases: IBM® MarketScan® Explorys® Claims-EMR Data Set (commercial) and OneFlorida Data Trust linked Medicaid-EHR. Positive and negative predictive values were calculated. Respectively, populations 1, 2, and 3 included 26,686, 26,095, and 2,564 episodes (commercial) and 29,117, 23,412, and 9,629 episodes (Florida Medicaid). The positive predictive value was >80% and the negative predictive value was >95% in each population, with the highest positive predictive value in population 3 (commercial: 91.9%; Medicaid: 93.1%). Findings did not vary substantially by patient age. Positive predictive values in populations 1 and 2 fluctuated during April–June 2020. They then stabilized in the commercial but not the Medicaid population. Negative predictive values were consistent over time in all populations and databases. Our findings indicate that U07.1 has high performance in identifying COVID-19 cases and noncases in claims databases. Performance may vary across populations and periods.

## Introduction

Coronavirus disease 2019 (COVID-19), caused by the novel strain of coronavirus called severe acute respiratory syndrome coronavirus 2 (SARS-CoV-2), has significantly affected the United States and countries throughout the world during the ongoing pandemic. A new diagnosis code for COVID-19 infections, U07.1, was introduced in the International Classification of Diseases, Tenth Revision, Clinical Modification (ICD-10-CM) on April 1, 2020 [1, 2]. ICD-10-CM coding guidelines specify that U07.1 should be used for a confirmed diagnosis based on a positive lab test or a provider’s documentation. In most real-world healthcare data, such as administrative claims, access to a provider’s notes and documentation is limited. Claims data enriched with linked electronic health records (EHRs), such as the data used in this study, include lab test results and thus allow for the assessment of the performance characteristics of the U07.1 code.

The Biologics Effectiveness and Safety (BEST) Initiative is an active surveillance program in the Food and Drug Administration’s Center for Biologics Evaluation and Research (CBER). This program contributes to CBER’s mission to evaluate and ensure the safety and effectiveness of biologic products, including vaccines. Understanding the performance characteristics of code U07.1 in administrative claims data sources to distinguish COVID-19 cases and noncases is important for conducting COVID-19 studies using such data, including postauthorization or postapproval evaluation of COVID-19 vaccine effectiveness.

Prior studies have evaluated the U07.1 code’s performance in identifying COVID-19 cases in hospital discharge data [3] but not in outpatient settings or administrative claims data. This study evaluated the performance characteristics of the ICD-10-CM code U07.1 in all settings among three care-seeking populations identified in the claims data. We used two linked claims-EHR data sources, consisting of commercially insured and Medicaid-insured individuals, respectively. In the claims portion of the linked data, we identified care-seekers and then classified them into COVID-19 cases and noncases on the basis of the presence or absence of the U07.1 code, respectively. The COVID-19 case status was verified by the severe acute respiratory syndrome coronavirus 2 (SARS-CoV-2) nucleic acid amplification test (NAAT) results in the EHR portion of the linked data. Within each study population, the positive predictive value (PPV) and negative predictive value (NPV) of U07.1 were estimated. PPV and NPV were also estimated monthly to evaluate temporal trends of the performance characteristics. These results will inform future observational studies in which the U07.1 code is used to identify COVID-19 cases and noncases in administrative claims data.

## Methods

### Data source

For the main analyses, we used two linked claims-EHR databases: IBM® MarketScan® Explorys® Claims-EMR (Electronic Medical Record) Data Set (CED) and OneFlorida Data Trust linked Medicaid-EHR. Because these databases contain deidentified data and are fully compliant with U.S. privacy laws and regulations (i.e., the Health Insurance Portability and Accountability Act), this study was exempt from institutional review board approval.

CED consists of deterministically linked claims (from the IBM MarketScan Commercial Database) and EHRs (from the IBM Explorys EHR Database) of the same individuals. The MarketScan Commercial Database contains data of commercially insured individuals from a selection of large employers, health plans, and government and public organizations. The Explorys EHR Database collects EHRs from more than 30 healthcare systems, spanning academic centers and community practices.

The OneFlorida Data Trust linked Medicaid-EHR contains claims data for Floridians enrolled in Medicaid and EHRs from public and private healthcare systems. Individuals are deterministically linked. EHRs came from integrated healthcare delivery networks, 13 large hospitals, and ambulatory care and primary care facilities.

To assess selection bias, we used the full MarketScan Commercial Database and OneFlorida Data Trust, the source claims databases from which the linked claims-EHR databases were created.

### Study period

The study period was April 1–December 31, 2020, for the commercially insured populations and April 1–November 30, 2020, for the Florida Medicaid populations.

### Study populations

#### Identify care episodes in claims portion of the claims-EHR linked data

We defined three populations of care episodes based on healthcare encounters in the claims portion of the linked data. A care episode identifies a cluster of events likely reflecting the continuum of care for an individual. One individual could have multiple care episodes. Population 1 consisted of episodes with a diagnosis of COVID-19, COVID-19-related symptoms, or suspected COVID-19 exposure (S1 Table 1 in S1 File). This list of codes includes ICD-10-CM diagnoses corresponding to the Centers for Disease Control and Prevention COVID-like illness definition (cough, fever, shortness of breath) [4] and additional codes that are potentially related to COVID-19 based on consultation with our physician consultants. The additional codes included COVID-19 diagnosis (U07.1; to capture cases without reported symptoms), other relevant conditions (e.g., respiratory failure) and symptoms (e.g., loss of smell and taste), and potential COVID-19 exposure. Population 2 consisted of episodes with a SARS-CoV-2 NAAT procedure code (S1 Table 2 in S1 File). Population 3 consisted of all-cause hospitalizations. Table 1 presents the criteria for grouping claims into episodes, including assignment of the index event (population 1: first encounter with a diagnosis of COVID-19, COVID-19-related symptoms, or suspected COVID-19 exposure; population 2: first NAAT procedure code; population 3: first all-cause hospitalization) and episode start and end dates (i.e., a date before the index date and after the last relevant encounter, respectively, to define a period during which NAAT results on the linked EHR are likely relevant). Only individuals aged <65 years at the index event with continuous coverage of medical benefits (allowing for a coverage gap up to 31 days) during the episodes and 6 months prior to the start of the episodes (baseline period) were retained.

**Table 1:** Steps to identify care episodes for three study populations from the linked claims-EHR databases.

Methods	Study population			Data used in linked claims-EHR
	Population 1	Population 2	Population 3	
**Identify events**	Diagnosis of COVID-19, COVID-19-related symptoms, or potential COVID-19 exposure in claims	SARS-CoV-2 NAAT procedures in claims	Acute all-cause hospitalization admissions in claims	Administrative claims portion of linked data
**Construct episodes**	Events were grouped into an episode when diagnosis dates are within 14 days of each other. An episode ends when there is a gap of >14 days between a diagnosis and any subsequent ones. When an episode extended into a hospitalization, the episode extended from the admission date to the discharge date.	Tests performed within 21 days of each other were grouped together as an episode. An episode ends when there is a gap of more than 21 days between a test and any subsequent ones.	Consecutive hospitalizations with a subsequent admission date within 1 day of the prior discharge date were connected to construct hospitalization episodes.	
**Define index event**	First encounter with a relevant symptom or diagnosis in an episode	First NAAT procedure in an episode	First admission date in an episode	
**Assign index date**	**Date of the index event**			
**Assign episode start date**	Index event date minus 7 days	Index event date minus 7 days	Index event date minus 14 days	
**Assign episode end date**	The date of the last event plus 7 days	The date of the last NAAT plus 14 days	Last discharge date	
**Limit to age <65 years**	Individuals must be <65 years of age on the index event date.			
**Require continuous enrollment**	Individuals must be continuously enrolled with medical benefits during the episode and during the 6-month baseline period prior to the episode. An enrollment gap of 31+ days was allowed.			
**Final study populations**	Limit to episodes with at least one observed positive or negative SARS-CoV-2 NAAT result in the linked EHRs during the episodes.			EHR portion of linked data

S1 Tables 1 and 2 in S1 File contain the code sets used to identify study populations 1 and 2, respectively

*EHR* electronic health record, *NAAT* nucleic acid amplification test, *SARS-CoV-2* severe acute respiratory syndrome coronavirus 2

#### Identify NAAT results in EHR portion of the claims-EHR linked data

Care episodes identified from the prior step were examined for SARS-CoV-2 NAAT results in the linked EHRs. Logical Observation Identifiers Names and Codes (LOINC) (S1 Table 3 in S1 File) were used to identify the SARS-CoV-2 NAAT results, and the observation date associated with the LOINC was the test result date. Episodes with at least one NAAT result during the episode were selected for the final three study populations (Table 1).

### U07.1 diagnosis status classification

We classified the study populations into U07.1-positive and U07.1-negative episodes based on the code’s presence or absence in claims during the episodes. Diagnoses from all care settings were used to classify study populations 1 and 2. Inpatient discharge diagnoses were used to classify study population 3.

### Performance characteristics

The PPV was the proportion of U07.1-positive episodes confirmed by a positive SARS-CoV-2 NAAT result in EHRs during the episode (when multiple NAAT results were present, one positive result was sufficient to confirm a positive case). The NPV was the proportion of U07.1-negative episodes confirmed by negative SARS-CoV-2 NAAT results in EHRs (when multiple NAAT results were present, all must be negative). PPV and NPV were calculated in each study population and stratified by calendar month, index care setting (inpatient, emergency department [ED], outpatient, and other settings—defined as index event occurring at an inpatient, ED, or outpatient setting or at a setting that is none of the above, respectively), and patient age at the index event. We calculated confidence intervals (CIs) for the PPVs and NPVs using the Agresti-Coull interval [5].

### Assessment of selection bias

Using the same methods that derived the study populations, we identified corresponding source populations in the full claims databases. We compared the source populations with the study populations for the distribution of patient demographics (e.g., age), clinical characteristics (e.g., Deyo-Charlson Comorbidity Index [CCI] [6, 7]), and healthcare utilization (e.g., ED visits). An absolute standardized mean difference (SMD) >0.20 was used as the threshold to denote potentially meaningful differences between the study and source populations.

## Results

### Population characteristics

**Fig 1:**
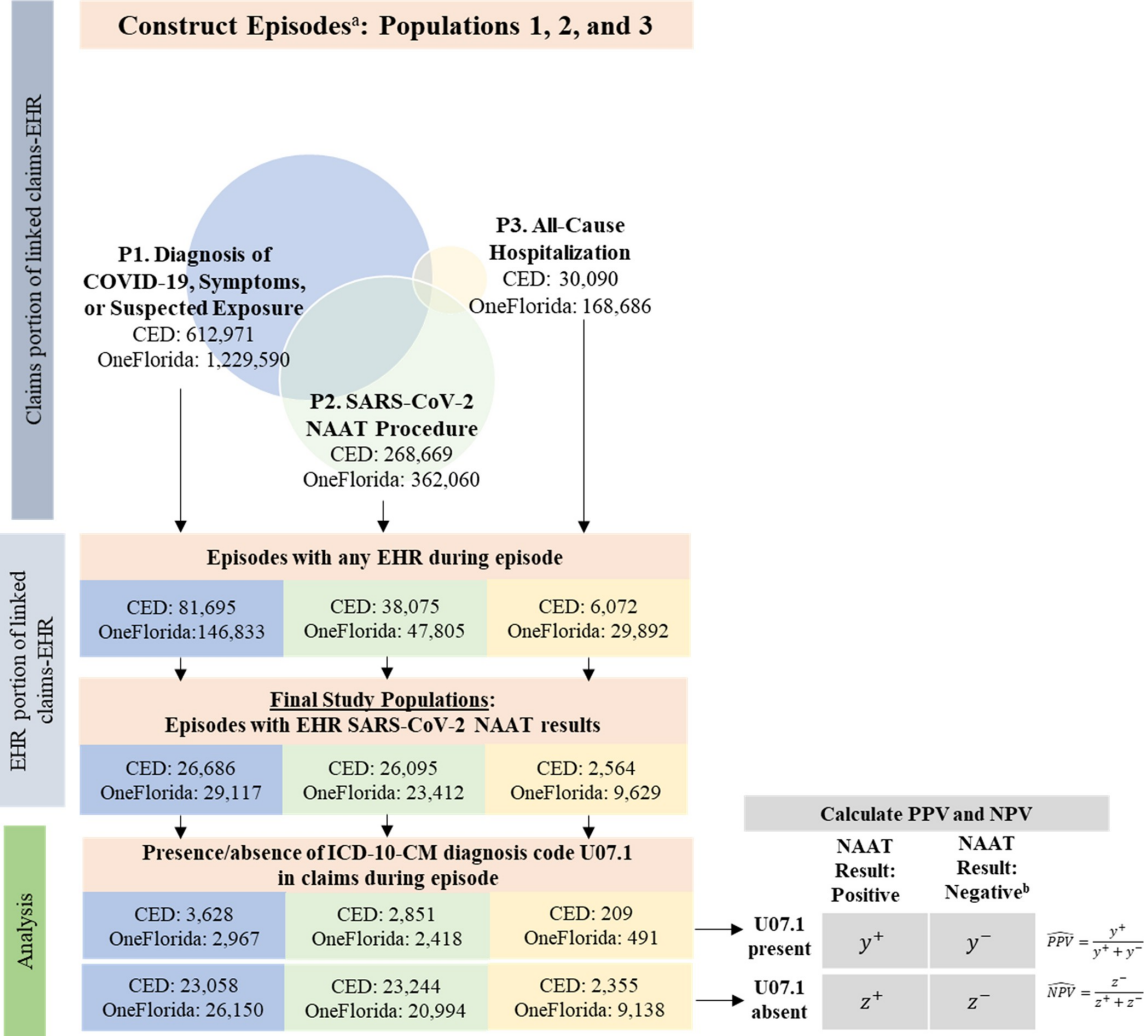
Study flow diagram to identify care episodes for study populations from the linked claims-EHR databases. *CED* IBM MarketScan Explorys Claims-EMR Data Set, *EHR* electronic health record, *ICD-10-CM* International Classification of Diseases, Tenth Revision, Clinical Modification, *NAAT* nucleic acid amplification test, *P* population, *SARS-CoV-2* severe acute respiratory syndrome coronavirus 2. ^a^ Episodes constructed in the claims portion of the linked claims-EHR data are of individuals aged <65 years at the index event with continuous coverage of medical benefits (allowing for a coverage gap up to 31 days) during the episodes and 6 months prior to the start of the episodes. ^b^ When multiple NAATs were present during the episode in the EHR, all must be negative. Data sources: IBM MarketScan Explorys Claims-EMR Data Set, April 1–December 31, 2020, and OneFlorida Data Trust linked Medicaid-EHR, April 1–November 30, 2020.

**Fig 2:**
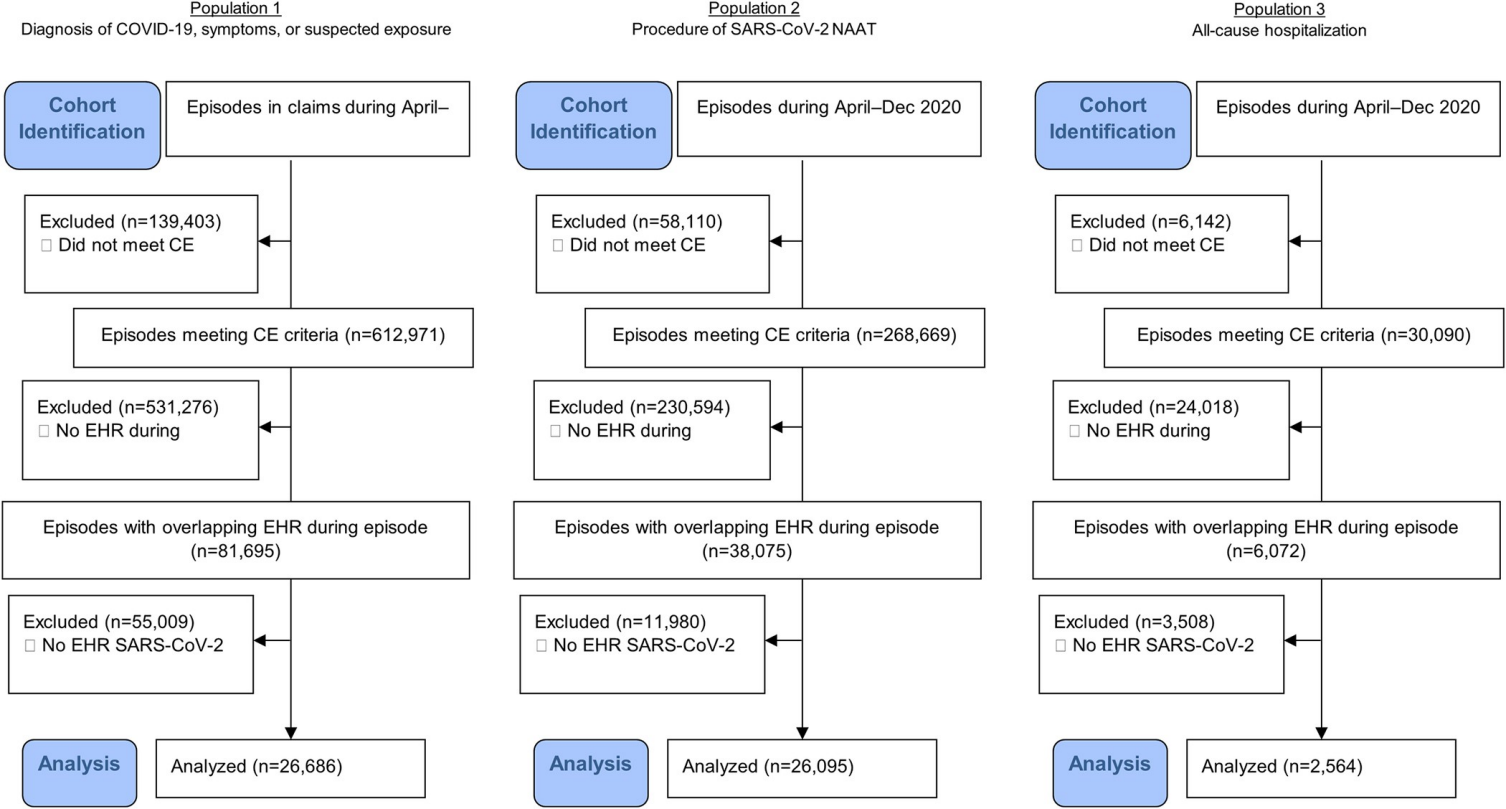
Cohort flow diagram for the commercially insured population in CED. CE criteria: Individuals must be continuously enrolled with medical benefits during the episode and during the 6-month baseline period prior to the episode. An enrollment gap of up to 31 days was allowed. *CE* continuous enrollment, *CED* IBM® MarketScan Explorys Claims-EMR Data Set, *EHR* electronic health record, *NAAT* nucleic acid amplification test, *SARS-CoV-2* severe acute respiratory syndrome coronavirus 2.

**Fig 3:**
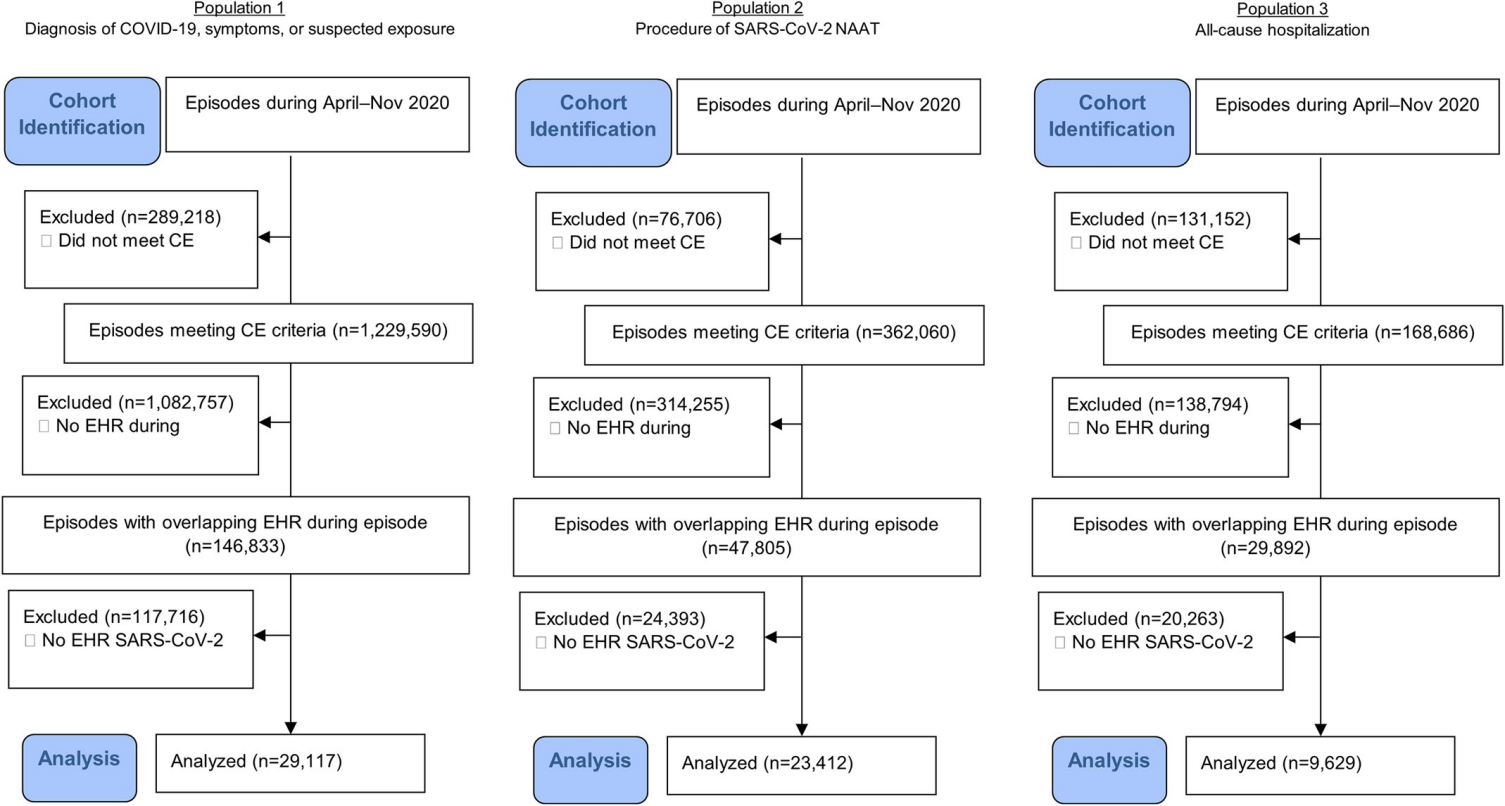
Cohort flow diagram for the Florida Medicaid population in OneFlorida. CE criteria: Individuals must be continuously enrolled with medical benefits during the episode and during the 6-month baseline period prior to the episode. An enrollment gap of up to 31 days was allowed. *CE* continuous enrollment, *EHR* electronic health record, *NAAT* nucleic acid amplification test, *SARS-CoV-2* severe acute respiratory syndrome coronavirus 2.

Fig 1 illustrates the identification of the study populations. The flow diagrams (Figs 2 and 3) further detail the steps taken to identify the subsets for analysis, including the number excluded and the reason for exclusion in each step, by database and population. Study populations from the commercially insured data consisted of 26,686 (population 1), 26,095 (population 2), and 2,564 (population 3) episodes, and study populations from Florida Medicaid consisted of 29,117 (population 1), 23,412 (population 2), and 9,629 (population 3) episodes (Table 2). Compared with populations 1 and 2, population 3 was older (mean age, commercial: 39 vs. 44 years; Florida Medicaid: 24–26 vs. 35 years). Populations 1 and 2 also had different clinical profiles from population 3. Certain potential COVID-19-related symptoms had higher proportions in populations 1 and 2 than in population 3, such as cough (commercial: 15.9–20.7% vs. 3.1%; Florida Medicaid: 14.2–14.6% vs. 1.0%) and fever (commercial: 9.7–12.3% vs. 6.7%; Florida Medicaid: 15.8–17.0% vs. 5.2%).

**Table 2:** Characteristics of the study populations.

Characteristic	Commercially insured (CED)			Florida Medicaid (OneFlorida)		
	Population 1: Diagnosis of COVID-19, symptoms, or potential exposure	Population 2: SARS-CoV-2 NAAT procedure in claims	Population 3: All-cause hospitalization	Population 1: Diagnosis of COVID-19, symptoms, or potential exposure	Population 2: SARS-CoV-2 NAAT procedure in claims	Population 3: All-cause hospitalization
**Total, N**	**26,686**	**26,095**	**2,564**	**29,117**	**23,412**	**9,629**
Male, %^a^	36.9	37.4	33.4	42.1	42.8	39.3
Age (years), mean, median, or %^a^						
Mean (SD)	39 (16)	39 (17)	44 (14)	26 (20)	24 (20)	35 (19)
Median (IQR)	40 (28,52)	40 (28,53)	45 (33,57)	21 (7,44)	18 (6,41)	34 (21,54)
0–17	11.5	12.2	2.3	44.6	49.4	19.7
18–25	9.1	8.9	6.2	9.4	9.0	13.0
26–35	19.7	18.8	25.7	12.4	11.4	19.0
36–45	19.9	20.0	17.0	9.9	9.5	12.4
46–55	21.2	21.4	21.6	10.0	9.0	14.6
56–64	18.6	18.9	27.2	13.6	11.8	21.3
Healthcare encounters during episode, %						
Inpatient stay ^b^	6.1	0.5	100.0	29.2	19.3	100.0
ICU stay ^c^	0.4	0.0	5.0	^—^ ^d^	^—^ ^d^	^—^ ^d^
ED visit ^e^	14.2	8.5	35.1	38.8	41.4	25.3
Potential COVID-19 symptoms, %						
Abdominal pain	7.4	1.5	11.6	16.5	13.7	4.6
Anorexia	0.2	0.1	0.4	0.9	0.7	0.4
Chest pain	5.9	1.8	10.2	12.6	10.1	5.3
Chills	2.2	1.8	0.2	0.5	0.4	0.1
Cough	20.7	15.9	3.1	14.6	14.2	1.0
Diarrhea	4.8	2.8	1.8	6.6	5.7	3.4
Dizziness	1.8	0.5	1.7	2.8	2.3	0.7
Fatigue	6.9	3.2	4.7	7.8	5.4	3.5
Fever	12.3	9.7	6.7	17.0	15.8	5.2
Headache	4.8	3.4	1.4	5.1	4.6	1.6
Loss of smell or taste	2.7	2.2	0.0	0.6	0.7	0.1
Myalgia	4.1	2.4	0.4	2.1	2.0	0.7
Nausea or vomiting	6.7	3.4	7.1	11.8	10.3	4.9
Palpitations	1.5	0.2	1.3	1.8	1.4	0.8
Shortness of breath	9.2	4.0	13.3	16.1	12.9	5.3
Sore throat	9.3	6.8	0.5	6.0	6.1	0.3
Potential COVID-19 complications, %						
Myocarditis/pericarditis	0.1	0.0	0.3	0.2	0.2	0.2
Multiorgan failure	0.2	0.0	2.3	2.7	1.5	5.5
Respiratory failure	1.1	0.1	9.1	6.6	4.0	14.3
Potential COVID-19 exposure^f^	76.0	68.1	38.7	85.2	84.0	74.8

*CED* IBM MarketScan Explorys Claims-EMR Data Set, *ED* emergency department, *ICU* intensive care unit, *IQR* interquartile range, *NAAT* nucleic acid amplification test, *SARS-CoV-2* severe acute respiratory syndrome coronavirus 2, *SD* standard deviation

^a^ Measured at the index event

^b^ Any hospitalization overlapping with the episode

^c^ Any hospitalization overlapping with the episode with revenue codes 0200–0209 during the stay

^d^ ICU stays could not be identified in OneFlorida Medicaid data due to lack of revenue codes

^e^ Any ED services during the episode

Data sources: IBM MarketScan Explorys Claims-EMR Data Set, April 1–December 31, 2020, and OneFlorida Data Trust linked Medicaid-EHR, April 1–November 30, 2020

Compared with Florida Medicaid, the study populations for the commercially insured had a lower proportion of individuals aged <18 years (commercial: 2.3% [population 3] to 12.2% [population 2]; Florida Medicaid: 19.7% [population 3] to 49.4% [population 2]). Study populations of the commercially insured were also less likely to have an ED visit during the episode (commercial: 8.5–35.1%; Florida Medicaid: 25.3–41.4%).

In both the commercially insured and Florida Medicaid populations, the distribution of NAATs as identified by LOINC codes was similar among study populations 1, 2, and 3 (S2 Table 1A and 1B in S2 File). The most common LOINC used in both databases was 94500–6 (SARS coronavirus 2 RNA [Presence] in Respiratory specimen by NAA with probe detection).

### Performance characteristics

**Fig 4:**
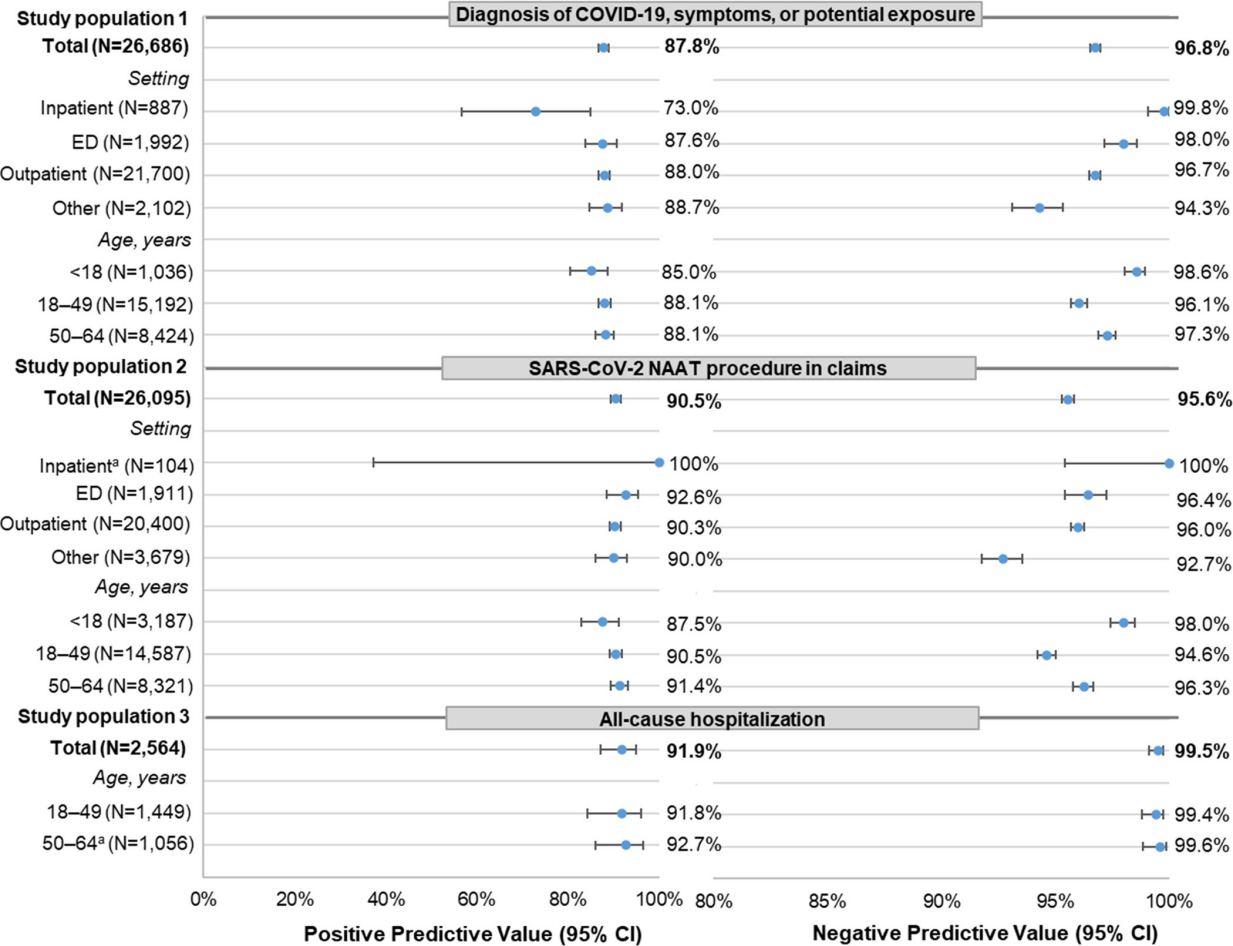
Performance characteristics (PPV/NPV) of diagnosis code U07.1 in claims data among commercially insured individuals. The PPV and NPV are not calculated for children <18 years old in study population 3 because the cell size was ≤1 in three cells of the 2X2 table for the calculation of PPV and NPV. Care setting and age were measured at the index event. Outpatient encounters include physician office, ambulatory care, outpatient hospital, and urgent care visits. Care setting was defined using a hierarchy if services at multiple settings were found on the index event date: inpatient, ED, outpatient, other. See S2 Table 2A in S2 File for the full results with exact CIs. The PPVs for study population 1 (inpatient setting at index event) and population 3 (all-cause hospitalization) are different because study population 1 episodes include diagnoses outside the hospitalization. *CED* IBM MarketScan Explorys Claims-EMR Data Set, *CI* confidence interval, *ED* emergency department, *ICD-10-CM* International Classification of Diseases, Tenth Revision, Clinical Modification, *NAAT* nucleic acid amplification test, *NPV* negative predictive value, *PPV* positive predictive value, *SARS-CoV-2* severe acute respiratory syndrome coronavirus 2. ^a^ Metric calculation used cell size <5. Data source: IBM MarketScan Explorys Claims-EMR Data Set, April 1–December 31, 2020.

**Fig 5:**
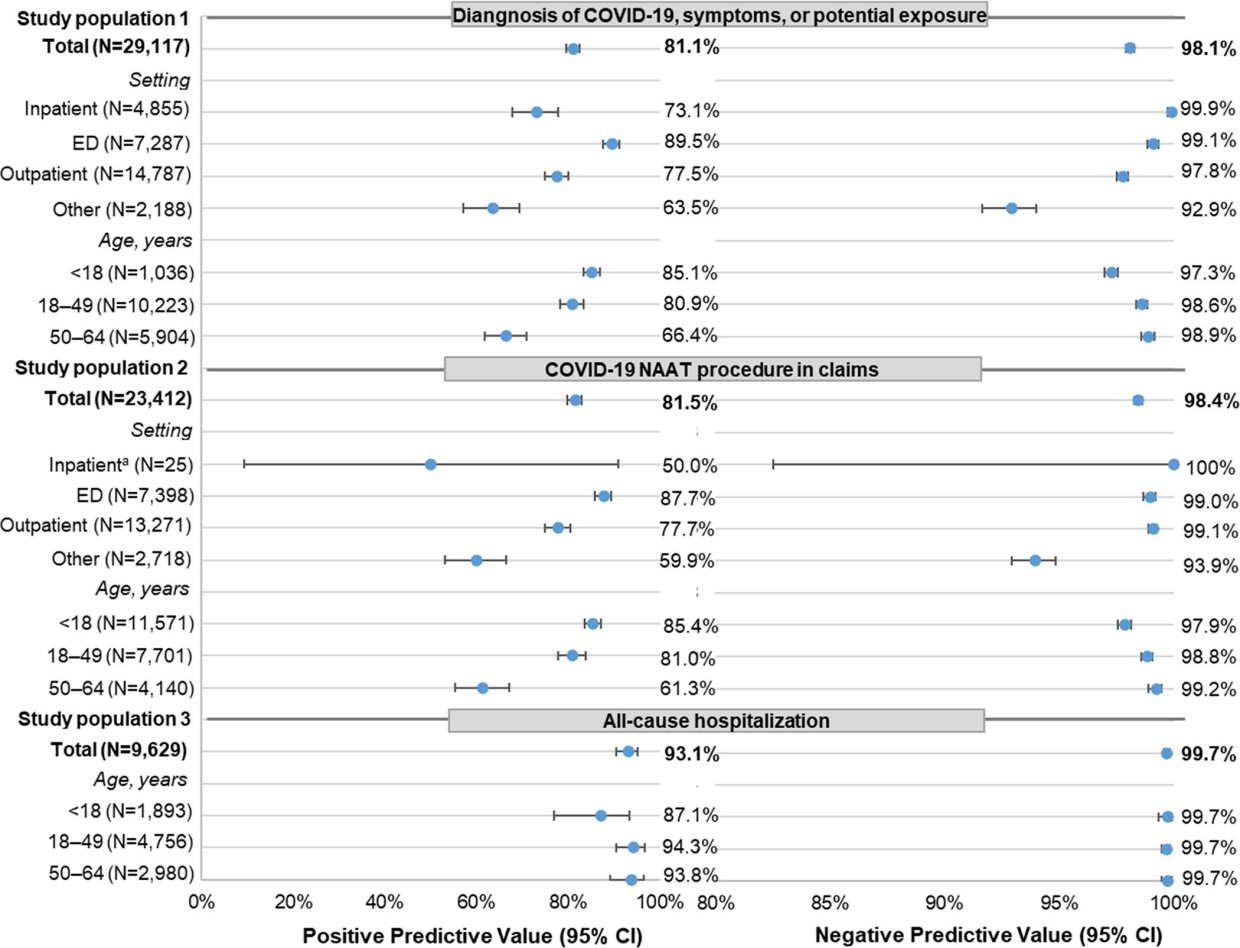
Performance characteristics (PPV/NPV) of diagnosis code U07.1 in claims data among individuals with Florida Medicaid. Outpatient encounters include all outpatient services because specific places of service (e.g., office, hospital outpatient) cannot be identified in the data. Care setting was defined using a hierarchy if services at multiple settings were found on the index event date: inpatient, ED, outpatient, other. See S2 Table 2B in S2 File for the full results with exact CIs. The PPVs for study population 1 (inpatient setting at index event) and population 3 (all-cause hospitalization) are different because study population 1 episodes include diagnoses outside the hospitalization. *CI* confidence interval, *ED* emergency department, *ICD-10-CM* International Classification of Diseases, Tenth Revision, Clinical Modification, *NAAT* nucleic acid amplification test, *PPV* positive predictive value, *SARS-CoV-2* severe acute respiratory syndrome coronavirus 2. ^a^ Metric calculation used cell size <5. Data source: OneFlorida Data Trust linked Medicaid-EHR, April 1–November 30, 2020.

The PPV of the COVID-19 diagnosis code U07.1 was above 80%, with narrow 95% CIs in each of the three study populations for the commercially insured and Florida Medicaid data (Figs 4 and 5; S2 Table 2A and 2B in S2 File). Population 3 had the highest PPV (commercial: 91.9%; Florida Medicaid: 93.1%). PPVs were similar in populations 1 and 2 but higher among individuals with commercial insurance (87.8% [population 1] and 90.5% [population 2]) than among Florida Medicaid enrollees (81.1% [population 1] and 81.5% [population 2]).

In both databases, the PPVs stratified by patient age and index care setting generally had narrow 95% CIs, except for the PPV from the inpatient subset of population 2 due to a small sample size. PPVs did not vary greatly by patient age in all three populations. However, PPVs showed variations by index care setting. In population 1, the PPVs were lower among episodes in an inpatient index care setting than those with an ED or outpatient setting for both the commercial (73.0% [inpatient] vs. 87.6% [ED] and 88.0% [outpatient]) and Medicaid (73.1% [inpatient] vs. 89.5% [ED] and 77.5% [outpatient]) databases. This, in part, was because some episodes with an index inpatient encounter had the U07.1 diagnosis at a noninpatient setting (commercial: 8 of 37; Florida Medicaid: 70 of 301). After limiting these episodes to those with a U07.1 diagnosis from the inpatient setting, PPVs increased to 89.7% (commercial) and 92.6% (Medicaid). In population 2, episodes with an inpatient index care setting were rare. The PPVs were similar between noninpatient settings among the commercially insured but were highest for the ED setting (87.7%) followed by outpatient (77.7%) and *other* (59.9%) settings among the Florida Medicaid population.

The NPV was high across all three study populations in both data sources with narrow 95% CIs. Populations 1 and 2 had similar NPVs (commercial: 96.8% and 95.6%, respectively; Medicaid: 98.1% and 98.4%, respectively). The NPV in population 3 was the highest (commercial: 99.5%; Medicaid: 99.7%). In both the commercial and Florida Medicaid data, the NPV did not vary greatly by patient age. By index care setting (populations 1 and 2), NPVs had similar patterns between the commercial and Florida Medicaid data: NPVs were high (>92%) across all index care settings, with the lowest values observed for episodes with an index care setting in the *other* category. Stratified NPVs also had narrow 95% CIs, except for the NPV of the inpatient subset of population 2 due to a small sample size.

### Monthly trend of performance characteristics

**Fig 6:**
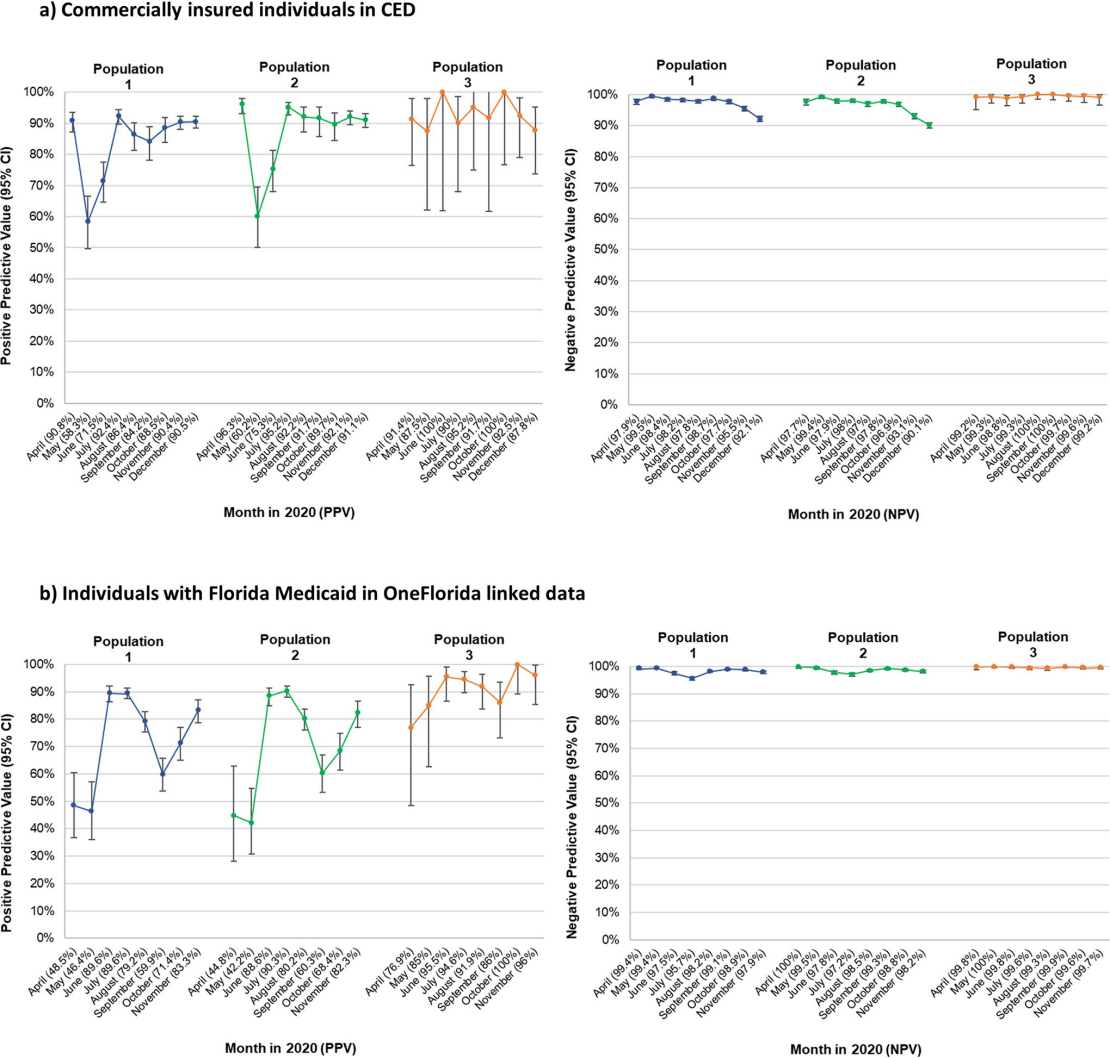
Monthly trends in performance characteristics (PPV/NPV) of diagnosis code U07.1 in claims data by population. Month corresponds to the month of the index event. Population 1: Diagnosis of COVID-19, symptoms, or potential exposure. Population 2: Severe acute respiratory syndrome coronavirus 2 nucleic acid amplification test procedure in claims. Population 3: All-cause hospitalization. *CED* IBM MarketScan Explorys Claims-EMR Data Set, *ICD-10-CM* International Classification of Diseases, Tenth Revision, Clinical Modification, *NPV* negative predictive value, *PPV* positive predictive value. Data sources: IBM MarketScan Explorys Claims-EMR Data Set, April 1–December 31, 2020, and OneFlorida Data Trust linked Medicaid-EHR, April 1–November 30, 2020.

Fig 6 displays PPVs and NPVs by month of index event (see S2 Table 3A and 3B in S2 File for underlying data). The 95% CIs were wide for the monthly PPVs but narrow for the NPVs, primarily due to the larger size of the monthly U07.1-negative episodes than the U07.1-positive episodes. For the commercially insured, PPVs in populations 1 and 2 showed a marked decrease early in the pandemic, around May–June 2020 (58.3–75.3%) but increased in July–December (population 1: 84.2–92.4%; population 2: 89.7–95.2%). PPVs were more stable in population 3 during April–December (ranging from 87.5 to 100.0%) despite the wide 95% CIs. In Florida Medicaid, PPVs fluctuated in populations 1 and 2 and, to a lesser extent, in population 3. In all populations, NPVs were consistently >90% over time.

### Assessment of selection bias

S2 Table 4A (commercially insured) and 4b (Florida Medicaid) in S2 File compare the source populations and the corresponding study populations to assess the potential selection bias introduced from subsetting source populations to the study populations with a linked EHR and a SARS-CoV-2 NAAT result.

Among both the commercially insured and Florida Medicaid enrollees, study populations generally resembled the source populations for most characteristics evaluated, with a few differences noted below.

Among the commercially insured, despite the fact that some baseline healthcare utilization measures differed slightly, the global comorbidity as measured by the CCI was similar between the study populations and source populations. Compared with the corresponding source populations, study populations 1 and 2 were more likely to have an outpatient index event (study vs. source, population 1: 81.3% vs. 71.8%; population 2: 78.2% vs. 40.1%), have a higher prevalence of certain symptoms (e.g., cough, population 1: 20.7% vs. 9.5%; population 2: 15.9% vs. 6.8%), and have a COVID-19 diagnosis (population 1: 13.6% vs. 6.1%; population 2: 10.9% vs. 5.2%). Episodes with suspected COVID-19 exposure diagnoses were overrepresented in study populations 1 and 3 compared with the source population (population 1: 76.0% vs. 57.0%; population 3: 38.7% vs. 25.1%) but underrepresented in population 2 (68.1% vs. 81.1%). Study population 3 was older than the corresponding source population (mean age: 43.8 vs. 40.7 years).

Among Florida Medicaid, compared with the corresponding source populations, mean baseline CCI was higher in study population 1 (0.8 vs. 0.5) and study population 2 (0.7 vs. 0.5) but similar in study population 3. Study population 1 was more likely to have a hospitalization during the baseline period than the corresponding source population (18.4% vs. 10.7%). However, all three study populations had similar clinical profiles for symptoms and potential COVID-19 complications as the source populations. Compared with the source population, study population 1 was more likely to have an inpatient index event (16.7% vs. 4.9%) and study population 2 was more likely to have an outpatient index event (56.7% vs. 37.1%). Study population 1 was more likely to have a COVID-19 diagnosis than the source population (10.2% vs. 4.1%). Like the commercially insured, Florida Medicaid episodes with suspected COVID-19 exposure diagnoses were overrepresented in study populations 1 and 3.

Overall, the study populations were generally representative of the source populations except for several characteristics noted above, which may potentially have introduced some selection bias.

## Discussion

Evidence suggests the ICD-10-CM U07.1 diagnosis code was broadly and rapidly adopted to identify COVID-19 patients in different care settings in the United States following its release on April 1, 2020 [8], but there is scant evidence regarding the performance characteristics of the diagnosis code in different care settings. We estimated the PPVs and NPVs of the COVID-19 diagnosis code U07.1 for three patient groups in the claims portion of two linked claims-EHR databases consisting of commercially insured and Medicaid-insured individuals. SARS-CoV-2 NAAT results from the EHR portion of the databases were used as the reference method. In each study population, the overall PPVs (>80%) and NPVs (>95%) were high and did not vary substantially by patient age but varied by healthcare setting.

For both the commercial insurance and Medicaid databases, PPVs and NPVs were similar in hospitalized individuals (population 3). They were also the highest in population 3, compared with the two other populations that were based on diagnosis of COVID-19, symptoms, or potential exposure (population 1) or NAAT procedure codes (population 2). PPVs and NPVs estimated in population 3 were consistent with other reported results for hospitalized patients: PPV: 91.3%, NPV: 99.8% (Kadri et al. [3]); PPV: 86% (Brown [9]); and PPV: 99.5%, NPV: 99.7% (Blatz et al. [10], which was among children). Our results and existing research indicate that the performance of the U07.1 code is higher in the inpatient setting. The PPVs and NPVs in populations 1 and 2 were similar and high for both the commercial insurance and Medicaid databases, although the PPV and NPV were both lower than those in population 3. The U07.1 code performance was overall poorer in Medicaid versus commercial insurance claims data.

For populations 1 and 2, PPVs were higher among populations with commercial insurance than those with Florida Medicaid. This may reflect that coding accuracy of COVID-19 is lower among Florida Medicaid than commercial insurance during the study period. However, many factors could contribute to this observed difference, such as different data processing systems or Florida’s local disease prevalence versus disease prevalence among the multiple geographic regions represented in the commercial insurance data.

PPVs varied by month, with greater variations in populations 1 and 2 and early in the pandemic. This instability lasted longer in the Medicaid data. Fluctuation of PPVs may be related to disease prevalence and coding practice changes. NPVs appeared stable in the study period for all populations evaluated.

Our study has several strengths. We used two large-scale linked claims-EHR databases representing commercially and publicly insured individuals. The use of linked claims-EHR databases to validate claims-based diagnoses is more efficient and cost-effective than the conventional medical chart review process. Additionally, NAAT results recorded on linked EHRs are the gold standard to ascertain COVID-19 case status, and as part of the structured EHR components, test results allow case adjudication to be fully automated. The BEST network brought together a broad range of real-world data, including linked claims-EHR data used in this study, which facilitated rapid assessment of the performance characteristics of the ICD-10-CM diagnosis code in the current public health crisis. We evaluated three care-seeking populations from which U07.1-positive and U07.1-negative cases were identified. The care-seeking U07.1-negative episodes allowed the estimation of NPVs and identified COVID-19 noncases that are comparable to the cases in terms of the type of care-seeking encounters. The study results showed the performance characteristics (PPV and NPV) of U07.1 were high in all three populations, which were selected based on different types of healthcare encounters with respect to COVID-19. Thus, the results may represent diverse care-seeking populations.

Our study has some limitations. A relatively small proportion of individuals in the administrative claims databases also had linked EHR data and were selected into the study populations. Additionally, EHR data came only from healthcare providers participating in the EHR network and thus may not include all the healthcare services received by the individuals in other healthcare facilities. Although the study populations were generally representative of the source populations for characteristics evaluated, unobserved characteristics may differ. This study used data from the pandemic’s early stages, and findings may not be generalizable to subsequent periods of the pandemic. The portability of performance characteristics of the U07.1 reported in this study depends on the prevalence of the disease in each population and characteristics of the databases.

## Conclusions

The BEST program allowed rapid validation of the COVID-19 ICD-10-CM diagnosis code using linked claims-EHR databases. When compared with NAAT results from EHRs, the overall PPV and NPV of the claims-based U07.1 code were high across all three study populations and did not vary significantly by age but varied by healthcare setting. In populations 1 and 2, PPVs varied by month and were lower for the Medicaid than the commercially insured population. NPVs were consistent over time in all populations and databases. This study demonstrates that the performance of the ICD-10-CM COVID-19 diagnosis code U07.1 in administrative claims databases is strong and that the code has the potential to be used in COVID-19 observational studies, including those of vaccine safety and effectiveness. This study, as a use case, demonstrated the utility of linked claims-EHR databases as well. Future efforts to link claims and EHR databases across healthcare systems can serve to create powerful tools to evaluate the safety and effectiveness of medical products in real-world settings and to contribute to public health.

## Supporting information

S1 File Clinical codes used to identify study populations. S1 Table 1 Codes used to identify study population 1 (diagnosis of COVID-19, symptoms, or potential exposure). S1 Table 2 Codes used to identify study population 2 (SARS-CoV-2 NAAT procedure in claims). S1 Table 3 Codes used to identify SARS-CoV-2 nucleic acid amplification test results in the linked electronic health records for study populations 1, 2, and 3.
(DOCX)

S2 File Additional results tables. S2 Table 1A Distribution of SARS-CoV-2 NAAT nucleic acid amplification test, in linked EHR data, by study population, among the commercially insured individuals in CED. S2 Table 1B Distribution of SARS-CoV-2 NAAT nucleic acid amplification test, in linked EHR data, by study population, among individuals with Florida Medicaid. S2 Table 2A (corresponding to Fig 4) Performance characteristics (PPV and NPV) of ICD-10-CM diagnosis code U07.1 in administrative claims data, by study population, care setting, and age at the index event, among the commercially insured individuals in CED. S2 Table 2B (corresponding to Fig 5) Performance characteristics of ICD-10-CM diagnosis code U07.1 in administrative claims data, by study population, care setting, and age at the index event, among individuals with Florida Medicaid in OneFlorida linked data. S2 Table 3A (corresponding to Fig 6) Monthly trends in performance characteristics (PPV/NPV) of diagnosis code U07.1 in claims data for commercially insured individuals in CED. S2 Table 3B (corresponding to Fig 6) Monthly trends in performance characteristics (PPV/NPV) of diagnosis code U07.1 in claims data for individuals with Florida Medicaid in OneFlorida linked data. S2 Table 4A Characteristics of study populations compared with the corresponding source populations, among individuals with commercial insurance. S2 Table 4B Characteristics of study populations compared with the corresponding source populations, among individuals with Florida Medicaid.
(DOCX)
