# Supplementary material for: Assessment of performance characteristics of COVID-19 ICD-10-CM diagnosis code U07.1 using SARS-CoV-2 nucleic acid amplification test results
Source: PLoS One. 2022 Aug 18;17(8):e0273196. doi: 10.1371/journal.pone.0273196 (PMC9387790; doi:10.1371/journal.pone.0273196)
Supplement: S1 File — S1 Table 1 Codes used to identify study population 1 (diagnosis of COVID-19, symptoms, or potential exposure). S1 Table 2 Codes used to identify study population 2 (SARS-CoV-2 NAAT procedure in claims). S1 Table 3 Codes used to identify SARS-CoV-2 nucleic acid amplification test results in the linked electronic health records for study populations 1, 2, and 3. (DOCX) [file pone.0273196.s001.docx]

**S1:** Clinical codes used to identify study populations

**S1 Table 1** Codes used to identify study population 1 (diagnosis of COVID-19, symptoms, or potential exposure)

| Code | Code description | Code type | OMOP concept ID | Symptom, exposure status, or condition |
| --- | --- | --- | --- | --- |
| R10.813 | Right lower quadrant abdominal tenderness | ICD-10-CM | 45544130 | abdominal_pain |
| R10.30 | Lower abdominal pain, unspecified | ICD-10-CM | 45563290 | abdominal_pain |
| R10.812 | Left upper quadrant abdominal tenderness | ICD-10-CM | 45534427 | abdominal_pain |
| R10.816 | Epigastric abdominal tenderness | ICD-10-CM | 45534428 | abdominal_pain |
| R10.827 | Generalized rebound abdominal tenderness | ICD-10-CM | 45548948 | abdominal_pain |
| R10.32 | Left lower quadrant pain | ICD-10-CM | 45553715 | abdominal_pain |
| R10.829 | Rebound abdominal tenderness, unspecified site | ICD-10-CM | 45553716 | abdominal_pain |
| R10.13 | Epigastric pain | ICD-10-CM | 45558454 | abdominal_pain |
| R10.11 | Right upper quadrant pain | ICD-10-CM | 45568112 | abdominal_pain |
| R10.9 | Unspecified abdominal pain | ICD-10-CM | 45568114 | abdominal_pain |
| R10.84 | Generalized abdominal pain | ICD-10-CM | 45558455 | abdominal_pain |
| R10.31 | Right lower quadrant pain | ICD-10-CM | 45582694 | abdominal_pain |
| R10.821 | Right upper quadrant rebound abdominal tenderness | ICD-10-CM | 45582695 | abdominal_pain |
| R10.12 | Left upper quadrant pain | ICD-10-CM | 45597168 | abdominal_pain |
| R10.824 | Left lower quadrant rebound abdominal tenderness | ICD-10-CM | 45602005 | abdominal_pain |
| R10.826 | Epigastric rebound abdominal tenderness | ICD-10-CM | 45602006 | abdominal_pain |
| R10.83 | Colic | ICD-10-CM | 45602007 | abdominal_pain |
| R10.814 | Left lower quadrant abdominal tenderness | ICD-10-CM | 45606795 | abdominal_pain |
| R10.33 | Periumbilical pain | ICD-10-CM | 45592407 | abdominal_pain |
| R10.815 | Periumbilic abdominal tenderness | ICD-10-CM | 45602004 | abdominal_pain |
| R10.822 | Left upper quadrant rebound abdominal tenderness | ICD-10-CM | 45597169 | abdominal_pain |
| R10.823 | Right lower quadrant rebound abdominal tenderness | ICD-10-CM | 45597170 | abdominal_pain |
| R10.811 | Right upper quadrant abdominal tenderness | ICD-10-CM | 45573009 | abdominal_pain |
| R10.10 | Upper abdominal pain, unspecified | ICD-10-CM | 45577781 | abdominal_pain |
| R10.817 | Generalized abdominal tenderness | ICD-10-CM | 45577782 | abdominal_pain |
| R10.825 | Periumbilic rebound abdominal tenderness | ICD-10-CM | 45577783 | abdominal_pain |
| R10.819 | Abdominal tenderness, unspecified site | ICD-10-CM | 45573010 | abdominal_pain |
| R10.0 | Acute abdomen | ICD-10-CM | 35211289 | abdominal_pain |
| R10.2 | Pelvic and perineal pain | ICD-10-CM | 35211290 | abdominal_pain |
| R63.0 | Anorexia | ICD-10-CM | 35211405 | anorexia |
| R07.1 | Chest pain on breathing | ICD-10-CM | 35211284 | chest_pain |
| R07.89 | Other chest pain | ICD-10-CM | 45602002 | chest_pain |
| R07.9 | Chest pain, unspecified | ICD-10-CM | 45534424 | chest_pain |
| R07.81 | Pleurodynia | ICD-10-CM | 45587497 | chest_pain |
| R07.82 | Intercostal pain | ICD-10-CM | 45597167 | chest_pain |
| R68.83 | Chills (without fever) | ICD-10-CM | 45577807 | chills |
| R05 | Cough | ICD-10-CM | 35211275 | cough* |
| R19.7 | Diarrhea, unspecified | ICD-10-CM | 45534435 | diarrhea |
| A08.39 | Other viral enteritis | ICD-10-CM | 45571414 | diarrhea |
| A08.4 | Viral intestinal infection, unspecified | ICD-10-CM | 35205449 | diarrhea |
| A09 | Infectious gastroenteritis and colitis, unspecified | ICD-10-CM | 35205450 | diarrhea |
| R42 | Dizziness and giddiness | ICD-10-CM | 35211350 | dizziness |
| R53.1 | Weakness | ICD-10-CM | 45602032 | fatigue |
| R53.81 | Other malaise | ICD-10-CM | 45582718 | fatigue |
| R53.83 | Other fatigue | ICD-10-CM | 45534458 | fatigue |
| R53.82 | Chronic fatigue, unspecified | ICD-10-CM | 45573032 | fatigue |
| R50.81 | Fever presenting with conditions classified elsewhere | ICD-10-CM | 45606818 | fever* |
| R50.9 | Fever, unspecified | ICD-10-CM | 35211387 | fever* |
| R50.82 | Postprocedural fever | ICD-10-CM | 45597189 | fever* |
| R51 | Headache | ICD-10-CM | 35211388 | headache |
| R43.0 | Anosmia | ICD-10-CM | 35211351 | loss_of_smell_taste |
| R43.9 | Unspecified disturbances of smell and taste | ICD-10-CM | 45573025 | loss_of_smell_taste |
| R43.1 | Parosmia | ICD-10-CM | 35211352 | loss_of_smell_taste |
| R43.2 | Parageusia | ICD-10-CM | 35211353 | loss_of_smell_taste |
| R43.8 | Other disturbances of smell and taste | ICD-10-CM | 35211354 | loss_of_smell_taste |
| R65.10 | Systemic inflammatory response syndrome (SIRS) of non-infectious origin without acute organ dysfunction | ICD-10-CM | 45539355 | multi_organ_failure |
| R65.11 | Systemic inflammatory response syndrome (SIRS) of non-infectious origin with acute organ dysfunction | ICD-10-CM | 45534462 | multi_organ_failure |
| R65.20 | Severe sepsis without septic shock | ICD-10-CM | 45548977 | multi_organ_failure |
| R65.21 | Severe sepsis with septic shock | ICD-10-CM | 45577803 | multi_organ_failure |
| M79.10 | Myalgia, unspecified site | ICD-10-CM | 1595617 | myalgia |
| M79.11 | Myalgia of mastication muscle | ICD-10-CM | 1595618 | myalgia |
| M79.12 | Myalgia of auxiliary muscles, head and neck | ICD-10-CM | 1595619 | myalgia |
| M79.18 | Myalgia, other site | ICD-10-CM | 1595620 | myalgia |
| I40.0 | Infective myocarditis | ICD-10-CM | 35207750 | myocarditis_pericarditis |
| I40.1 | Isolated myocarditis | ICD-10-CM | 35207751 | myocarditis_pericarditis |
| I40.8 | Other acute myocarditis | ICD-10-CM | 35207752 | myocarditis_pericarditis |
| I40.9 | Acute myocarditis, unspecified | ICD-10-CM | 35207753 | myocarditis_pericarditis |
| I51.4 | Myocarditis, unspecified | ICD-10-CM | 35207798 | myocarditis_pericarditis |
| J10.82 | Influenza due to other identified influenza virus with myocarditis | ICD-10-CM | 45591546 | myocarditis_pericarditis |
| J11.82 | Influenza due to unidentified influenza virus with myocarditis | ICD-10-CM | 45543255 | myocarditis_pericarditis |
| B33.22 | Viral myocarditis | ICD-10-CM | 45581156 | myocarditis_pericarditis |
| B33.23 | Viral pericarditis | ICD-10-CM | 45571465 | myocarditis_pericarditis |
| I30.0 | Acute nonspecific idiopathic pericarditis | ICD-10-CM | 35207715 | myocarditis_pericarditis |
| I30.1 | Infective pericarditis | ICD-10-CM | 35207716 | myocarditis_pericarditis |
| I30.8 | Other forms of acute pericarditis | ICD-10-CM | 35207717 | myocarditis_pericarditis |
| I30.9 | Acute pericarditis, unspecified | ICD-10-CM | 35207718 | myocarditis_pericarditis |
| I32 | Pericarditis in diseases classified elsewhere | ICD-10-CM | 35207725 | myocarditis_pericarditis |
| I41 | Myocarditis in diseases classified elsewhere | ICD-10-CM | 35207754 | myocarditis_pericarditis |
| I31.0 | Chronic adhesive pericarditis | ICD-10-CM | 35207719 | myocarditis_pericarditis |
| I31.1 | Chronic constrictive pericarditis | ICD-10-CM | 35207720 | myocarditis_pericarditis |
| R11.0 | Nausea | ICD-10-CM | 45534429 | nausea_vomiting |
| R11.10 | Vomiting, unspecified | ICD-10-CM | 45602008 | nausea_vomiting |
| R11.11 | Vomiting without nausea | ICD-10-CM | 45573011 | nausea_vomiting |
| R11.12 | Projectile vomiting | ICD-10-CM | 45558456 | nausea_vomiting |
| R11.13 | Vomiting of fecal matter | ICD-10-CM | 45573012 | nausea_vomiting |
| R11.14 | Bilious vomiting | ICD-10-CM | 45568115 | nausea_vomiting |
| R11.15 | Cyclical vomiting syndrome unrelated to migraine | ICD-10-CM | 1553844 | nausea_vomiting |
| R11.2 | Nausea with vomiting, unspecified | ICD-10-CM | 45606796 | nausea_vomiting |
| R11.3 | Other vomiting without nausea | ICD-10-CM | 35211291 | nausea_vomiting |
| R00.2 | Palpitations | ICD-10-CM | 35211263 | palpitations |
| R00.8 | Other abnormalities of heart beat | ICD-10-CM | 35211264 | palpitations |
| R00.9 | Unspecified abnormalities of heart beat | ICD-10-CM | 45577776 | palpitations |
| J95.821 | Acute postprocedural respiratory failure | ICD-10-CM | 45538487 | respiratory_failure |
| J95.822 | Acute and chronic postprocedural respiratory failure | ICD-10-CM | 45562471 | respiratory_failure |
| J96.92 | Respiratory failure, unspecified with hypercapnia | ICD-10-CM | 45533563 | respiratory_failure |
| J96.21 | Acute and chronic respiratory failure with hypoxia | ICD-10-CM | 45543283 | respiratory_failure |
| J96.11 | Chronic respiratory failure with hypoxia | ICD-10-CM | 45538489 | respiratory_failure |
| J96.00 | Acute respiratory failure, unspecified whether with hypoxia or hypercapnia | ICD-10-CM | 45605906 | respiratory_failure |
| J96.10 | Chronic respiratory failure, unspecified whether with hypoxia or hypercapnia | ICD-10-CM | 45548131 | respiratory_failure |
| J96.01 | Acute respiratory failure with hypoxia | ICD-10-CM | 45567283 | respiratory_failure |
| J96.90 | Respiratory failure, unspecified, unspecified whether with hypoxia or hypercapnia | ICD-10-CM | 45567284 | respiratory_failure |
| J96.22 | Acute and chronic respiratory failure with hypercapnia | ICD-10-CM | 45581868 | respiratory_failure |
| J96.91 | Respiratory failure, unspecified with hypoxia | ICD-10-CM | 45605907 | respiratory_failure |
| J96.12 | Chronic respiratory failure with hypercapnia | ICD-10-CM | 45572177 | respiratory_failure |
| J96.02 | Acute respiratory failure with hypercapnia | ICD-10-CM | 45596289 | respiratory_failure |
| J96.20 | Acute and chronic respiratory failure, unspecified whether with hypoxia or hypercapnia | ICD-10-CM | 45596290 | respiratory_failure |
| P28.5 | Respiratory failure of newborn | ICD-10-CM | 35210515 | respiratory_failure |
| R09.2 | Respiratory arrest | ICD-10-CM | 35211287 | respiratory_failure |
| R06.09 | Other forms of dyspnea | ICD-10-CM | 45548944 | shortness_of_breath* |
| R06.00 | Dyspnea, unspecified | ICD-10-CM | 45587496 | shortness_of_breath* |
| R06.01 | Orthopnea | ICD-10-CM | 45597165 | shortness_of_breath* |
| R06.02 | Shortness of breath | ICD-10-CM | 45534422 | shortness_of_breath* |
| R06.03 | Acute respiratory distress | ICD-10-CM | 1326788 | shortness_of_breath* |
| R06.1 | Stridor | ICD-10-CM | 35211276 | shortness_of_breath* |
| R06.2 | Wheezing | ICD-10-CM | 35211277 | shortness_of_breath* |
| R06.4 | Hyperventilation | ICD-10-CM | 35211279 | shortness_of_breath* |
| R06.5 | Mouth breathing | ICD-10-CM | 35211280 | shortness_of_breath* |
| R06.82 | Tachypnea, not elsewhere classified | ICD-10-CM | 45539314 | shortness_of_breath* |
| J02.8 | Acute pharyngitis due to other specified organisms | ICD-10-CM | 35207923 | sore_throat |
| J02.9 | Acute pharyngitis, unspecified | ICD-10-CM | 35207924 | sore_throat |
| U07.1 | COVID-19, virus identified | ICD-10-CM | 702953 | COVID-19 |
| Z03.818 | Encounter for observation for suspected exposure to other biological agents ruled out | ICD-10-CM | 45552061 | COVID-19_exposure |
| Z20.828 | Contact with and (suspected) exposure to other viral communicable diseases | ICD-10-CM | 45542411 | COVID-19_exposure |
| Z11.59 | Encounter for screening for other viral diseases | ICD-10-CM | 45595484 | COVID-19_exposure |

*ICD-10-CM* International Classification of Diseases, Tenth Revision, Clinical Modification, *OMOP* Observational Medical Outcomes Partnership

*Denotes diagnoses that are included in the Centers for Disease Control and Prevention COVID-like illness definition

**S1 Table 2** Codes used to identify study population 2 (SARS-CoV-2 NAAT procedure in claims)

| HCPCS/CPT | Description |
| --- | --- |
| 87635 | Infectious agent detection by nucleic acid (DNA or RNA); severe acute respiratory syndrome coronavirus 2 (SARS-CoV-2) Coronavirus disease [COVID-19]), amplified probe technique |
| U0001 | CDC testing laboratories to test patients for SARS-CoV-2 |
| U0002 | Non-CDC laboratory tests for SARS-CoV-2/2019-nCoV (COVID-19) |
| U0003 | Infectious agent detection by nucleic acid (DNA or RNA); severe acute respiratory syndrome coronavirus 2 (SARS-CoV-2) (Coronavirus disease [COVID-19]), amplified probe technique |
| U0004 | 2019-nCoV Coronavirus, SARS-CoV-2/2019-nCoV (COVID-19), any technique, multiple types or subtypes (includes all targets), non-CDC |
| 0202U | Infectious disease (bacterial or viral respiratory tract infection), pathogen-specific nucleic acid (DNA or RNA), 22 targets including severe acute respiratory syndrome coronavirus 2 (SARS-CoV-2), qualitative RT-PCR, nasopharyngeal swab |
| 0223U | Infectious disease (bacterial or viral respiratory tract infection), pathogen-specific nucleic acid (DNA or RNA), 22 targets including severe acute respiratory syndrome coronavirus 2 (SARS-CoV-2), qualitative RT-PCR, nasopharyngeal swab |

*CDC* Centers for Disease Control and Prevention, *CPT* Current Procedural Terminology, *HCPCS* Healthcare Common Procedure Coding System, *NAAT* nucleic acid amplification test, *RT-PCR* reverse transcription polymerase chain reaction, *SARS-CoV-2* severe acute respiratory syndrome coronavirus 2

**S1 Table 3** Codes used to identify SARS-CoV-2 NAAT results in the linked electronic health records for study populations 1, 2, and 3

| LOINC | Long common name |
| --- | --- |
| 94745-7 | SARS-CoV-2 (COVID-19) RNA [Cycle Threshold #] in Respiratory specimen by NAA with probe detection |
| 94746-5 | SARS-CoV-2 (COVID-19) RNA [Cycle Threshold #] in Unspecified specimen by NAA with probe detection |
| 94819-0 | SARS-CoV-2 (COVID-19) RNA [Log #/volume] (viral load) in Unspecified specimen by NAA with probe detection |
| 94565-9 | SARS coronavirus 2 RNA [Presence] in Nasopharynx by NAA with non-probe detection |
| 94759-8 | SARS-CoV-2 (COVID-19) RNA [Presence] in Nasopharynx by NAA with probe detection |
| 94500-6 | SARS coronavirus 2 RNA [Presence] in Respiratory specimen by NAA with probe detection |
| 94845-5 | SARS-CoV-2 (COVID-19) RNA [Presence] in Saliva (oral fluid) by NAA with probe detection |
| 94660-8 | SARS-CoV-2 (COVID-19) RNA [Presence] in Serum or Plasma by NAA with probe detection |
| 94309-2 | SARS Coronavirus 2 RNA [Presence] in Unspecified specimen Qualitative by NAA with probe detection |
| 41458-1 | SARS coronavirus RNA [Presence] in Unspecified specimen by NAA with probe detection |
| 94534-5 | SARS coronavirus 2 RdRp gene [Presence] in Respiratory specimen by NAA with probe detection |
| 95608-6 | SARS-CoV-2 (COVID-19) RNA [Presence] in Respiratory specimen by NAA with non-probe detection |
| 94533-7 | SARS-CoV-2 (COVID19) N gene [Presence] in Respiratory specimen by NAA with probe detection |
| 94640-0 | SARS coronavirus 2 S gene [Presence] in Respiratory specimen by NAA with probe detection |
| 94559-2 | SARS coronavirus 2 ORF1ab region [Presence] in Respiratory specimen by NAA with probe detection |
| 94502-2 | SARS-related coronavirus RNA [Presence] in Respiratory specimen by NAA with probe detection |
| 95423-0 | Influenza virus A + B and SARS-CoV-2 (COVID-19) identified in Respiratory specimen by NAA with probe detection |
| 95409-9 | SARS coronavirus 2 (COVID19) N gene [Presence] in Nose by NAA with probe detection |
| 95425-5 | SARS-CoV-2 (COVID-19) N gene [Presence] in Saliva (oral fluid) by NAA with probe detection |
| 94760-6 | SARS coronavirus 2 N gene [Presence] in Nasopharynx by NAA with probe detection |
| 95406-5 | SARS-CoV-2 (COVID19) RNA [Presence] in Nose by NAA with probe detection |
| 94758-0 | SARS-related coronavirus E gene [Presence] in Respiratory specimen by NAA with probe detection |
| 96091-4 | SARS-CoV-2 (COVID-19) RdRp gene [Presence] in Saliva (oral fluid) by NAA with probe detection |
| 94316-7 | SARS-CoV-2 (COVID-19) N gene [Presence] in Specimen by NAA with probe detection |

*SARS-CoV-2* severe acute respiratory syndrome coronavirus 2, *NAAT nucleic acid amplification test, LOINC* Logical Observation Identifiers Names and Codes, *NAA* nucleic acid amplification

Source: Centers for Diseases Control and Prevention. LOINC in vitro diagnostic (LIVD) test code mapping for SARS-CoV-2 tests. <https://www.cdc.gov/csels/dls/sars-cov-2-livd-codes.html>. Accessed 1 Apr 2021.
