# Supplementary material for: Assessment of performance characteristics of COVID-19 ICD-10-CM diagnosis code U07.1 using SARS-CoV-2 nucleic acid amplification test results
Source: PLoS One. 2022 Aug 18;17(8):e0273196. doi: 10.1371/journal.pone.0273196 (PMC9387790; doi:10.1371/journal.pone.0273196)
Supplement: S2 File — S2 Table 1A Distribution of SARS-CoV-2 NAAT nucleic acid amplification test, in linked EHR data, by study population, among the commercially insured individuals in CED. S2 Table 1B Distribution of SARS-CoV-2 NAAT nucleic acid amplification test, in linked EHR data, by study population, among individuals with Florida Medicaid. S2 Table 2A (corresponding to Fig 4) Performance characteristics (PPV and NPV) of ICD-10-CM diagnosis code U07.1 in administrative claims data, by study population, care setting, and age at the index event, among the commercially insured individuals in CED. S2 Table 2B (corresponding to Fig 5) Performance characteristics of ICD-10-CM diagnosis code U07.1 in administrative claims data, by study population, care setting, and age at the index event, among individuals with Florida Medicaid in OneFlorida linked data. S2 Table 3A (corresponding to Fig 6) Monthly trends in performance characteristics (PPV/NPV) of diagnosis code U07.1 in claims data for commercially insured individuals in CED. S2 Table 3B (corresponding to Fig 6) Monthly trends in performance characteristics (PPV/NPV) of diagnosis code U07.1 in claims data for individuals with Florida Medicaid in OneFlorida linked data. S2 Table 4A Characteristics of study populations compared with the corresponding source populations, among individuals with commercial insurance. S2 Table 4B Characteristics of study populations compared with the corresponding source populations, among individuals with Florida Medicaid. (DOCX) [file pone.0273196.s002.docx]

**S2:** Additional results tables

**S2 Table 1a** Distribution of SARS-CoV-2 NAAT nucleic acid amplification test, in linked EHR data, by study population, among the commercially insured individuals in CED

| **NAAT LOINC** | **Description** | **Population 1 (N=26,686)** | **Population 2 (N=26,095)** | **Population 3 (N=2,564)** |
| --- | --- | --- | --- | --- |
|  |  | n (%) | | |
| 94309-2 | SARS Coronavirus 2 RNA [Presence] in Unspecified specimen Qualitative by NAA with probe detection | 28 (0.1%) | 34 (0.1%) | 2 (0.1%) |
| 94500-6 | SARS coronavirus 2 RNA [Presence] in Respiratory specimen by NAA with probe detection | 26,566 (99.6%) | 26,005 (99.7%) | 2,561 (99.9%) |
| 94502-2 | SARS-related coronavirus RNA [Presence] in Respiratory specimen by NAA with probe detection | 31 (0.1%) | 30 (0.1%) | 0 (0.0%) |
| 94534-5 | SARS coronavirus 2 RdRp gene [Presence] in Respiratory specimen by NAA with probe detection | 113 (0.4%) | 77 (0.3%) | 4 (0.1%) |

*Column percentages sum up to be greater than 100% because episodes may have multiple tests with different LOINC code.

*CED* IBM MarketScan Explorys Claims-EMR Data Set, *EHR* electronic health record, *LOINC* Logical Observation Identifiers Names and Codes, *NAAT* nucleic acid amplification test, *SARS-CoV-2* severe acute respiratory syndrome coronavirus 2

**S2 Table 1b** Distribution of SARS-CoV-2 NAAT nucleic acid amplification test, in linked EHR data, by study population, among individuals with Florida Medicaid

| **NAAT LOINC** | **Description** | **Population 1 (N=29,117)** | **Population 2 (N=23,412)** | **Population 3 (N=9,629)** |
| --- | --- | --- | --- | --- |
|  |  | n (%) | | |
| 48767-8 | SARS coronavirus RNA [Presence] in Unspecified specimen by NAA with probe detection | 1664 (6.3%) | 1478 (6.3%) | 401 (4.2%) |
| 94309-2 | SARS Coronavirus 2 RNA [Presence] in Unspecified specimen Qualitative by NAA with probe detection | 4869 (16.5%) | 3871 (16.5%) | 2273 (23.6%) |
| 94316-7 | SARS-CoV-2 (COVID-19) N gene [Presence] in Specimen by NAA with probe detection | 134 (0.2%) | 54 (0.2%) | 63 (0.7%) |
| 94500-6 | SARS coronavirus 2 RNA [Presence] in Respiratory specimen by NAA with probe detection | 23106 (81.9%) | 19171 (81.9%) | 6391 (66.4%) |
| 94534-5 | SARS coronavirus 2 RdRp gene [Presence] in Respiratory specimen by NAA with probe detection | 1407 (2.3%) | 546 (2.3%) | 1153 (12%) |

*Column percentages sum up to be greater than 100% because episodes may have multiple tests with different LOINC code.

*EHR* electronic health record, *LOINC* Logical Observation Identifiers Names and Codes, *NAAT* nucleic acid amplification test, *SARS-CoV-2* severe acute respiratory syndrome coronavirus 2

**S2 Table 2a (corresponding to Fig 4)** Performance characteristics (PPV and NPV) of ICD-10-CM diagnosis code U07.1 in administrative claims data, by study population, care setting, and age at the index event, among the commercially insured individuals in CED

| Subgroup | Positive predictive value (95% CI) | | | Negative predictive value (95% CI) | | |
| --- | --- | --- | --- | --- | --- | --- |
|  | Population 1: Diagnosis of COVID-19, symptoms, or potential exposure | Population 2:  SARS-CoV-2 NAAT procedure in claims | Population 3:  All-cause hospitalization | Population 1: Diagnosis of COVID-19, symptoms, or potential exposure | Population 2:  SARS-CoV-2 NAAT procedure in claims | Population 3:  All-cause hospitalization |
| **Overall** | 87.8 (86.7, 88.9) | 90.5 (89.3, 91.5) | 91.9 (87.2, 95.0) | 96.8 (96.5, 97.0) | 95.6 (95.3, 95.8) | 99.5 (99.1, 99.7) |
| Care setting^a^ |  |  |  |  |  |  |
| Inpatient | 73.0 (56.5, 84.9) | 100 (37.3, 105.6) | 91.9 (87.2, 95.0) | 99.8 (99.1, 100.0) | 100.0 (95.4, 100.0) | 91.9 (87.2, 95.0) |
| ED | 87.6 (83.7, 90.7) | 92.6 (88.5, 95.3) | N/A | 98.0 (97.1, 98.6) | 96.4 (95.4, 97.2) | N/A |
| Outpatient | 88.0 (86.7, 89.1) | 90.3 (89.0, 91.5) | N/A | 96.7 (96.5, 97.0) | 96.0 (95.7, 96.3) | N/A |
| Other | 88.7 (84.7, 91.8) | 90.0 (86.0, 93.0) | N/A | 94.3 (93.1, 95.3) | 92.7 (91.7, 93.5) | N/A |
| Age, years^a^ |  |  |  |  |  |  |
| <18 | 85.0 (80.4, 88.7) | 87.5 (82.9, 91.0) | —^b^ | 98.6 (98.0, 98.9) | 98.0 (97.4, 98.5) | —^b^ |
| 18–49 | 88.1 (86.6, 89.4) | 90.5 (89.0, 91.8) | 91.8 (84.2, 96.0) | 96.1 (95.7, 96.4) | 94.6 (94.2, 95.0) | 99.4 (98.8, 99.7) |
| 50–64 | 88.1 (86.0, 89.9) | 91.4 (89.3, 93.2) | 92.7 (85.9, 96.5) | 97.3 (96.9, 97.7) | 96.3 (95.8, 96.7) | 99.6 (98.9, 99.9) |

*CED* IBM MarketScan Explorys Claims-EMR Data Set, *CI* confidence interval, *ED* emergency department, *N/A* not applicable, *NAAT* nucleic acid amplification test, *SARS-CoV-2* severe acute respiratory syndrome coronavirus 2

^a^ Measured at the index event. Outpatient encounters include physician office, ambulatory care, and urgent care visits. Care setting was defined using a hierarchy if multiple settings were found at the index event: inpatient, ED, outpatient, telemedicine, other.

^b^ Not calculated because the cell size was ≤1 in three cells of the 2X2 table for the calculation of positive predictive value and negative predictive value

Data source: IBM MarketScan Explorys Claims-EMR Data Set, April 1–December 31, 2020

**S2 Table 2b (corresponding to Fig 5)** Performance characteristics of ICD-10-CM diagnosis code U07.1 in administrative claims data, by study population, care setting, and age at the index event, among individuals with Florida Medicaid in OneFlorida linked data

| Subgroup | Positive predictive value (95% CI) | | | Negative predictive value (95% CI) | | |
| --- | --- | --- | --- | --- | --- | --- |
|  | Population 1: Diagnosis of COVID-19, symptoms, or potential exposure | Population 2:  SARS-CoV-2 NAAT procedure in claims | Population 3:  All-cause hospitalization | Population 1: Diagnosis of COVID-19, symptoms, or potential exposure | Population 2:  SARS-CoV-2 NAAT procedure in claims | Population 3:  All-cause hospitalization |
| **Overall** | 81.1 (79.6, 82.5) | 81.5 (79.8, 83) | 93.1 (90.4, 95.1) | 98.1 (97.9, 98.3) | 98.4 (98.3, 98.6) | 99.7 (99.6, 99.8) |
| Care setting^a^ |  |  |  |  |  |  |
| Inpatient | 73.1 (67.7, 77.9) | 50.0 (9.2, 90.8) | 93.1 (90.4, 95.1) | 99.9 (99.7, 100.0) | 100.0 (82.5, 100.0) | 99.7 (99.6, 99.8) |
| ED | 89.5 (87.6, 91.1) | 87.7 (85.8, 89.4) | N/A | 99.1 (98.8, 99.3) | 99.0 (98.7, 99.2) | N/A |
| Outpatient | 77.5 (75.0, 79.9) | 77.7 (74.8, 80.4) | N/A | 97.8 (97.5, 98.0) | 99.1 (98.9, 99.2) | N/A |
| Other | 63.5 (57.1, 69.4) | 59.9 (53.0, 66.4) | N/A | 92.9 (91.7, 94.0) | 93.9 (92.9, 94.8) | N/A |
| Age, years^a^ |  |  |  |  |  |  |
| <18 | 85.1 (83.3, 86.8) | 85.4 (83.5, 87.2) | 87.1 (76.9, 93.4) | 97.3 (97.0, 97.6) | 97.9 (97.6, 98.1) | 99.7 (99.3, 99.9) |
| 18–49 | 80.9 (78.1, 83.4) | 81.0 (77.8, 83.8) | 94.3 (90.3, 96.8) | 98.6 (98.3, 98.8) | 98.8 (98.6, 99.1) | 99.7 (99.4, 99.8) |
| 50–64 | 66.4 (61.8, 70.8) | 61.3 (55.3, 67.0) | 93.8 (89.2, 96.5) | 98.9 (98.6, 99.1) | 99.2 (98.9, 99.5) | 99.7 (99.5, 99.9) |

*CI* confidence interval, *ED* emergency department, *N/A* not applicable, *NAAT* nucleic acid amplification test, *SARS-CoV-2* severe acute respiratory syndrome coronavirus 2

^a^ Measured at the index event. Outpatient encounters include physician office, ambulatory care, and urgent care visits. Care setting was defined using a hierarchy if multiple settings were found at the index event: inpatient, ED, outpatient, telemedicine, other.

Data source: OneFlorida Data Trust linked Medicaid-EHR, April 1–November 30, 2020.

**S2 Table 3a (corresponding to Fig 6)** Monthly trends in performance characteristics (PPV/NPV) of diagnosis code U07.1 in claims data for commercially insured individuals in CED

| **Month** | **PPV** | **PPV 95% CI lower bound** | **PPV 95% CI upper bound** | **NPV** | **NPV 95% CI lower bound** | **NPV 95% CI upper bound** |
| --- | --- | --- | --- | --- | --- | --- |
| **Study population 1** | | | | | | |
| 2020-04 | 90.8 | 87.3 | 93.5 | 97.9 | 96.9 | 98.6 |
| 2020-05 | 58.3 | 49.6 | 66.6 | 99.5 | 99.0 | 99.8 |
| 2020-06 | 71.5 | 64.7 | 77.4 | 98.4 | 97.8 | 98.9 |
| 2020-07 | 92.4 | 89.8 | 94.4 | 98.2 | 97.7 | 98.6 |
| 2020-08 | 86.4 | 81.4 | 90.3 | 97.8 | 97.2 | 98.4 |
| 2020-09 | 84.2 | 78.2 | 88.8 | 98.7 | 98.2 | 99.1 |
| 2020-10 | 88.5 | 83.9 | 91.9 | 97.7 | 97.0 | 98.2 |
| 2020-11 | 90.4 | 88.1 | 92.3 | 95.5 | 94.7 | 96.1 |
| 2020-12 | 90.5 | 88.4 | 92.3 | 92.1 | 91.2 | 92.9 |
| Overall | 87.8 | 86.7 | 88.9 | 96.8 | 96.5 | 97.0 |
| **Study population 2** | | | | | | |
| 2020-04 | 96.3 | 93.1 | 98.1 | 97.7 | 96.6 | 98.4 |
| 2020-05 | 60.2 | 50.1 | 69.5 | 99.4 | 98.8 | 99.7 |
| 2020-06 | 75.3 | 68.1 | 81.3 | 97.9 | 97.2 | 98.5 |
| 2020-07 | 95.2 | 92.7 | 96.8 | 98.0 | 97.4 | 98.4 |
| 2020-08 | 92.2 | 87.3 | 95.3 | 97.0 | 96.3 | 97.7 |
| 2020-09 | 91.7 | 85.7 | 95.4 | 97.8 | 97.1 | 98.4 |
| 2020-10 | 89.7 | 84.5 | 93.4 | 96.9 | 96.2 | 97.5 |
| 2020-11 | 92.1 | 89.6 | 94.0 | 93.1 | 92.2 | 93.8 |
| 2020-12 | 91.1 | 88.7 | 93.1 | 90.1 | 89.1 | 91.0 |
| Overall | 90.5 | 89.3 | 91.5 | 95.6 | 95.3 | 95.8 |
| **Study population 3** | | | | | | |
| 2020-04 | 91.4 | 76.5 | 97.9 | 99.2 | 95.2 | 100.3 |
| 2020-05 | 87.5 | 62.1 | 97.9 | 99.3 | 97.3 | 100.0 |
| 2020-06 | 100.0 | 61.8 | 104.8 | 98.8 | 96.4 | 99.8 |
| 2020-07 | 90.0 | 68.1 | 98.5 | 99.3 | 97.2 | 100.0 |
| 2020-08 | 95.2 | 75.0 | 101.0 | 100.0 | 98.6 | 100.0 |
| 2020-09 | 91.7 | 61.7 | 100.8 | 100.0 | 98.3 | 100.0 |
| 2020-10 | 100.0 | 76.6 | 103.4 | 99.7 | 97.8 | 100.2 |
| 2020-11 | 92.5 | 79.1 | 98.2 | 99.6 | 97.5 | 100.2 |
| 2020-12 | 87.8 | 73.6 | 95.3 | 99.2 | 96.7 | 100.0 |
| Overall | 91.9 | 87.2 | 95.0 | 99.5 | 99.1 | 99.7 |

*CED* IBM MarketScan Claims-EMR Data Set, *CI* confidence interval, *NPV* negative predictive value, *PPV* positive predictive value

Data source: IBM MarketScan Explorys Claims-EMR Data Set (study population). April 1–December 31, 2020.

**S2 Table 3b (corresponding to Fig 6)** Monthly trends in performance characteristics (PPV/NPV) of diagnosis code U07.1 in claims data for individuals with Florida Medicaid in OneFlorida linked data

| **Month** | **PPV** | **PPV 95% CI lower bound** | **PPV 95% CI upper bound** | **NPV** | **NPV 95% CI lower bound** | **NPV 95% CI upper bound** |
| --- | --- | --- | --- | --- | --- | --- |
| **Study population 1** | | | | | | |
| 2020-04 | 48.5 | 36.8 | 60.4 | 99.4 | 98.8 | 99.8 |
| 2020-05 | 46.4 | 36.0 | 57.2 | 99.4 | 99.0 | 99.7 |
| 2020-06 | 89.6 | 86.3 | 92.1 | 97.5 | 96.9 | 98.0 |
| 2020-07 | 89.6 | 87.5 | 91.3 | 95.7 | 95.0 | 96.3 |
| 2020-08 | 79.2 | 75.3 | 82.7 | 98.2 | 97.8 | 98.6 |
| 2020-09 | 59.9 | 53.7 | 65.8 | 99.1 | 98.7 | 99.3 |
| 2020-10 | 71.4 | 64.9 | 77.0 | 98.9 | 98.5 | 99.1 |
| 2020-11 | 83.3 | 78.8 | 87.0 | 97.9 | 97.4 | 98.4 |
| Overall | 81.1 | 79.6 | 82.5 | 98.1 | 97.9 | 98.3 |
| **Study population 2** | | | | | | |
| 2020-04 | 44.8 | 28.1 | 62.8 | 100.0 | 99.3 | 100.0 |
| 2020-05 | 42.2 | 30.7 | 54.6 | 99.5 | 99.0 | 99.8 |
| 2020-06 | 88.6 | 84.9 | 91.5 | 97.8 | 97.2 | 98.3 |
| 2020-07 | 90.3 | 88.1 | 92.1 | 97.2 | 96.5 | 97.7 |
| 2020-08 | 80.2 | 76.0 | 83.8 | 98.5 | 98.0 | 98.9 |
| 2020-09 | 60.3 | 53.2 | 67.0 | 99.3 | 98.9 | 99.5 |
| 2020-10 | 68.4 | 61.3 | 74.7 | 98.8 | 98.4 | 99.1 |
| 2020-11 | 82.3 | 77.0 | 86.7 | 98.2 | 97.6 | 98.6 |
| Overall | 81.5 | 79.8 | 83.0 | 98.4 | 98.3 | 98.6 |
| **Study population 3** | | | | | | |
| 2020-04 | 76.9 | 48.5 | 92.7 | 99.8 | 98.9 | 100.1 |
| 2020-05 | 85.0 | 62.6 | 95.7 | 100.0 | 99.6 | 100.0 |
| 2020-06 | 95.5 | 86.7 | 99.0 | 99.8 | 99.3 | 100.0 |
| 2020-07 | 94.6 | 89.8 | 97.3 | 99.6 | 98.9 | 99.8 |
| 2020-08 | 91.9 | 83.7 | 96.3 | 99.3 | 98.7 | 100.0 |
| 2020-09 | 86.0 | 73.2 | 93.5 | 99.9 | 99.5 | 100.0 |
| 2020-10 | 100.0 | 89.2 | 101.7 | 99.6 | 99.1 | 99.9 |
| 2020-11 | 96.0 | 85.5 | 99.7 | 99.7 | 99.1 | 99.9 |
| Overall | 93.1 | 90.4 | 95.1 | 99.7 | 99.6 | 99.8 |

*CI* confidence interval, *NPV* negative predictive value, *PPV* positive predictive value

Data source: OneFlorida Data Trust linked Medicaid-EHR, April 1–November 30, 2020.

**S2 Table** **4a** Characteristics of study populations compared with the corresponding source populations, among individuals with commercial insurance

| Characteristic | Population 1: Diagnosis of COVID-19, symptoms, or potential exposure | | | Population 2: SARS-CoV-2 NAAT procedure in claims | | | Population 3: All-cause hospitalization | | |
| --- | --- | --- | --- | --- | --- | --- | --- | --- | --- |
|  | Source population,  %, mean | Study population | | Source population,  %, mean | Study population | | Source population,  %, mean | Study population | |
|  |  | %, mean | SMD |  | %, mean | SMD |  | %, mean | SMD |
| **Total, N** | **10,901,160** | **26,686** | **N/A** | **4,859,504** | **26,095** | **N/A** | **431,662** | **2,564** | **N/A** |
| Male, %^a^ | 40.4 | 36.9 | –0.07 | 42.7 | 37.4 | –0.11 | 34.8 | 33.4 | –0.03 |
| Age, mean years^a^ | 38.2 | 38.9 | 0.04 | 36.8 | 38.8 | 0.12 | 40.7 | 43.8 | **0.21** |
| Age group, years, %^a^ |  |  |  |  |  |  |  |  |  |
| 0–17 | 12.4 | 11.5 | –0.03 | 13.0 | 12.2 | –0.02 | 6.3 | 2.3 | –0.20 |
| 18–25 | 14.3 | 9.1 | –0.16 | 16.3 | 8.9 | **–0.22** | 11.3 | 6.2 | –0.18 |
| 26–35 | 16.1 | 19.7 | 0.09 | 17.6 | 18.8 | 0.03 | 24.7 | 25.7 | 0.02 |
| 36–45 | 17.9 | 19.9 | 0.05 | 17.3 | 20.0 | 0.07 | 15.9 | 17.0 | 0.03 |
| 46–55 | 20.6 | 21.2 | 0.01 | 19.1 | 21.4 | 0.06 | 17.9 | 21.6 | 0.09 |
| 56–64 | 18.8 | 18.6 | 0.00 | 16.7 | 18.9 | 0.06 | 24.0 | 27.2 | 0.07 |
| Care setting at index event, %^a,b^ |  |  |  |  |  |  |  |  |  |
| Inpatient | 1.1 | 3.3 | 0.15 | 0.4 | 0.4 | 0.01 | 100.0 | 100.00 | 0.00 |
| ED | 5.9 | 7.5 | 0.06 | 3.6 | 7.3 | 0.16 | 0.0 | 0.0 | 0.00 |
| Outpatient^c^ | 71.8 | 81.3 | **0.23** | 40.1 | 78.2 | **0.84** | 0.0 | 0.0 | 0.00 |
| Other | 21.3 | 7.9 | **–0.39** | 56.0 | 14.1 | **–0.98** | 0.0 | 0.0 | 0.00 |
| Length of hospital stay, mean days | N/A | N/A | N/A | N/A | N/A | N/A | 5.2 | 5.7 | 0.06 |
| Episode length, mean days | 17.5 | 18.3 | 0.09 | 23.6 | 23.6 | –0.01 | 19.5 | 20.1 | 0.07 |
| Healthcare encounters during episode, % |  |  |  |  |  |  |  |  |  |
| Inpatient stay | 2.1 | 6.1 | **0.20** | 0.4 | 0.5 | 0.00 | 100.0 | 100.0 | 0.00 |
| ICU stay | 0.2 | 0.4 | 0.05 | 0.0 | 0.0 |  | 3.6 | 5.0 | 0.07 |
| ED visit | 9.0 | 14.2 | 0.16 | 4.2 | 8.5 | 0.18 | 30.0 | 35.1 | 0.11 |
| Potential COVID-19 symptoms during the episode, % |  |  |  |  |  |  |  |  |  |
| Abdominal pain | 12.0 | 7.4 | –0.16 | 0.7 | 1.5 | 0.08 | 12.2 | 11.6 | –0.02 |
| Anorexia | 0.2 | 0.2 | 0.00 | 0.0 | 0.1 | 0.02 | 0.3 | 0.4 | 0.01 |
| Chest pain | 6.3 | 5.9 | –0.02 | 0.8 | 1.8 | 0.09 | 8.8 | 10.2 | 0.05 |
| Chills | 0.4 | 2.2 | 0.16 | 0.3 | 1.8 | 0.14 | 0.1 | 0.2 | 0.01 |
| Cough | 9.5 | 20.7 | **0.31** | 6.8 | 15.9 | **0.29** | 3.2 | 3.1 | 0.00 |
| Diarrhea | 3.3 | 4.8 | 0.08 | 1.0 | 2.8 | 0.13 | 2.4 | 1.8 | –0.05 |
| Dizziness | 3.0 | 1.8 | –0.07 | 0.2 | 0.5 | 0.05 | 1.6 | 1.7 | 0.01 |
| Fatigue | 9.9 | 6.9 | –0.11 | 1.5 | 3.2 | 0.12 | 4.2 | 4.7 | 0.03 |
| Fever | 5.1 | 12.3 | **0.26** | 4.1 | 9.7 | **0.22** | 6.0 | 6.7 | 0.03 |
| Headache | 3.5 | 4.8 | 0.06 | 0.9 | 3.4 | 0.17 | 2.0 | 1.4 | –0.05 |
| Loss of smell or taste | 0.7 | 2.7 | 0.16 | 0.5 | 2.2 | 0.15 | 0.1 | 0.0 | –0.01 |
| Myalgia | 3.3 | 4.1 | 0.04 | 0.7 | 2.4 | 0.14 | 0.4 | 0.4 | 0.01 |
| Nausea or vomiting | 4.7 | 6.7 | 0.08 | 1.0 | 3.4 | 0.16 | 6.9 | 7.1 | 0.01 |
| Palpitations | 3.1 | 1.5 | –0.11 | 0.1 | 0.2 | 0.04 | 0.9 | 1.3 | 0.04 |
| Shortness of breath | 6.6 | 9.2 | 0.10 | 1.8 | 4.0 | 0.13 | 11.6 | 13.3 | 0.05 |
| Sore throat | 5.9 | 9.3 | 0.13 | 3.3 | 6.8 | 0.16 | 0.3 | 0.5 | 0.04 |
| Potential COVID-19 complications during the episode, % |  |  |  |  |  |  |  |  |  |
| Myocarditis/pericarditis | 0.1 | 0.1 | 0.00 | 0.0 | 0.0 | 0.00 | 0.3 | 0.3 | 0.00 |
| Respiratory failure | 0.7 | 1.1 | 0.04 | 0.1 | 0.1 | 0.00 | 8.6 | 9.1 | 0.01 |
| Multiorgan failure | 0.1 | 0.2 | 0.02 | 0.0 | 0.0 | 0.00 | 2.4 | 2.3 | 0.00 |
| COVID-19 diagnosis (U07.1) | 6.1 | 13.6 | **0.25** | 5.2 | 10.9 | **0.21** | 6.9 | 8.2 | 0.05 |
| Potential COVID-19 exposure | 57.0 | 76.0 | **0.41** | 81.1 | 68.1 | **–0.30** | 25.1 | 38.7 | **0.30** |
| Episode overlapped with pregnancy, % | 0.9 | 1.9 | 0.08 | 0.9 | 1.0 | 0.01 | 24.0 | 20.7 | –0.08 |
| Baseline characteristics^d^ |  |  |  |  |  |  |  |  |  |
| CCI, mean score | 0.4 | 0.5 | 0.08 | 0.3 | 0.5 | 0.12 | 1.1 | 1.3 | 0.12 |
| Inpatient stay, % | 3.2 | 4.4 | 0.06 | 2.7 | 4.1 | 0.08 | 16.2 | 18.5 | 0.06 |
| ED visit, % | 12.2 | 15.5 | 0.10 | 10.4 | 15.2 | 0.14 | 28.3 | 33.4 | 0.11 |
| Outpatient^c^ visits, mean N | 5.5 | 7.1 | 0.19 | 5.0 | 7.0 | **0.26** | 10.1 | 12.1 | 0.16 |
| Prescription fills, mean N | 5.0 | 3.2 | **–0.27** | 4.4 | 3.3 | –0.17 | 7.2 | 6.0 | –0.13 |

Bold red font indicates absolute (SMD) of 0.2 or more

*CCI* Charlson Comorbidity Index, *ED* emergency department, *ICU* intensive care unit, *N/A* not applicable, *NAAT* nucleic acid amplification test, *SARS-CoV-2* severe acute respiratory syndrome coronavirus 2, *SMD* standardized mean difference

^a^ Measured at the index event

^b^ Care setting was defined using a hierarchy if multiple settings were found at the index event: inpatient, ED, outpatient, telemedicine, other

^c^ Outpatient encounters include physician office, ambulatory care, and urgent care visits

^d^ Measured across all claims during the 6 months prior to the start of the episode

Data source: IBM MarketScan Commercial Database (source population); IBM MarketScan Explorys Claims-EMR Data Set (study population). April 1–December 31, 2020.

**S2 Table 4b** Characteristics of study populations compared with the corresponding source populations, among individuals with Florida Medicaid

| Characteristic | Population 1: Diagnosis of COVID-19, symptoms, or potential exposure | | | Population 2: SARS-CoV-2 NAAT in claims | | | Population 3: All-cause hospitalization | | |
| --- | --- | --- | --- | --- | --- | --- | --- | --- | --- |
|  | Source population, %, mean | Study population | | Source population, %, mean | Study population | | Source population, %, mean | Study population | |
|  |  | %, mean | SMD |  | %, mean | SMD |  | %, mean | SMD |
| **Total, N** | **1,229,590** | **29,117** | **N/A** | **362,060** | **23,412** | **N/A** | **168,686** | **9,629** | **N/A** |
| Male, %^a^ | 37.9 | 42.1 | 0.09 | 40.9 | 42.8 | 0.04 | 34.9 | 39.3 | 0.09 |
| Age, mean years^a^ | 24.7 | 26.1 | 0.07 | 22.6 | 24.2 | 0.09 | 35.4 | 35.5 | 0.00 |
| Age group, years, %^a^ |  |  |  |  |  |  |  |  |  |
| 0–17 | 46.7 | 44.6 | –0.04 | 51.7 | 49.4 | –0.05 | 15.6 | 19.7 | 0.11 |
| 18–25 | 10.6 | 9.4 | –0.04 | 10.8 | 9.0 | –0.06 | 16.3 | 13.0 | –0.09 |
| 26–35 | 12.9 | 12.4 | –0.01 | 12.3 | 11.4 | –0.03 | 22.9 | 19.0 | –0.10 |
| 36–45 | 10.0 | 9.9 | 0.00 | 8.8 | 9.5 | 0.02 | 13.0 | 12.4 | –0.02 |
| 46–55 | 8.7 | 10.0 | 0.04 | 7.3 | 9.0 | 0.06 | 13.1 | 14.6 | 0.04 |
| 56–64 | 11.1 | 13.6 | 0.08 | 9.1 | 11.8 | 0.09 | 19.1 | 21.3 | 0.05 |
| Care setting at index event, %^a,b^ |  |  |  |  |  |  |  |  |  |
| Inpatient | 4.9 | 16.7 | **0.39** | 0.1 | 0.1 | 0.02 | 100.0 | 100.0 | 0.00 |
| ED | 19.5 | 25.0 | 0.13 | 26.9 | 31.6 | 0.10 | 0.0 | 0.0 | 0.00 |
| Outpatient^c^ | 57.0 | 50.8 | –0.13 | 37.1 | 56.7 | **0.40** | 0.0 | 0.0 | 0.00 |
| Other | 18.5 | 7.5 | **–0.33** | 35.9 | 11.6 | **–0.60** | 0.0 | 0.0 | 0.00 |
| Length of hospital stay, mean days | N/A | N/A | N/A | N/A | N/A | N/A | 5.1 | 6.7 | 0.17 |
| Episode length, mean days | 16.9 | 21.3 | 0.30 | 22.5 | 22.9 | 0.06 | 19.1 | 20.7 | 0.17 |
| Healthcare encounters during episode, % |  |  |  |  |  |  |  |  |  |
| Inpatient stay | 9.1 | 29.2 | 0.53 | 10.8 | 19.3 | 0.24 | 100.0 | 100.0 | 0.00 |
| ED visit | 30.0 | 38.8 | 0.19 | 33.4 | 41.4 | 0.17 | 22.7 | 25.3 | 0.06 |
| Potential COVID-19 symptoms during the episode, % |  |  |  |  |  |  |  |  |  |
| Abdominal pain | 20.8 | 16.5 | –0.11 | 9.2 | 13.7 | 0.14 | 4.4 | 4.6 | 0.01 |
| Anorexia | 1.2 | 0.9 | –0.03 | 0.4 | 0.7 | 0.03 | 0.4 | 0.4 | 0.00 |
| Chest pain | 9.4 | 12.6 | 0.10 | 6.7 | 10.1 | 0.12 | 3.7 | 5.3 | 0.07 |
| Chills | 0.3 | 0.5 | 0.04 | 0.4 | 0.4 | 0.01 | 0.0 | 0.1 | 0.02 |
| Cough | 13.6 | 14.6 | 0.03 | 15.0 | 14.2 | –0.02 | 0.7 | 1.0 | 0.04 |
| Diarrhea | 5.6 | 6.6 | 0.04 | 4.1 | 5.7 | 0.08 | 1.8 | 3.4 | 0.10 |
| Dizziness | 3.2 | 2.8 | –0.02 | 1.5 | 2.3 | 0.06 | 0.6 | 0.7 | 0.02 |
| Fatigue | 8.2 | 7.8 | –0.02 | 4.1 | 5.4 | 0.06 | 2.4 | 3.5 | 0.07 |
| Fever | 11.9 | 17.0 | 0.15 | 14.5 | 15.8 | 0.04 | 2.6 | 5.2 | 0.14 |
| Headache | 6.0 | 5.1 | –0.04 | 3.5 | 4.6 | 0.05 | 1.1 | 1.6 | 0.04 |
| Loss of smell or taste | 0.3 | 0.6 | 0.04 | 0.6 | 0.7 | 0.01 | 0.0 | 0.1 | 0.02 |
| Myalgia | 1.6 | 2.1 | 0.04 | 1.3 | 2.0 | 0.06 | 0.3 | 0.7 | 0.07 |
| Nausea or vomiting | 10.5 | 11.8 | 0.04 | 6.9 | 10.3 | 0.12 | 3.0 | 4.9 | 0.10 |
| Palpitations | 2.3 | 1.8 | –0.04 | 0.8 | 1.4 | 0.06 | 0.3 | 0.8 | 0.06 |
| Shortness of breath | 10.2 | 16.1 | 0.17 | 9.0 | 12.9 | 0.12 | 3.7 | 5.3 | 0.08 |
| Sore throat | 7.8 | 6.0 | –0.07 | 6.7 | 6.1 | –0.03 | 0.2 | 0.3 | 0.01 |
| Potential COVID-19 complications during the episode, % |  |  |  |  |  |  |  |  |  |
| Myocarditis/pericarditis | 0.1 | 0.2 | 0.04 | 0.1 | 0.2 | 0.03 | 0.1 | 0.2 | 0.03 |
| Multiorgan failure | 0.7 | 2.7 | 0.16 | 0.7 | 1.5 | 0.08 | 3.2 | 5.5 | 0.11 |
| Respiratory failure | 2.7 | 6.6 | 0.18 | 2.0 | 4.0 | 0.12 | 8.8 | 14.3 | 0.17 |
| COVID-19 diagnosis (U07.1) | 4.1 | 10.2 | **0.24** | 8.4 | 10.3 | 0.07 | 4.1 | 5.1 | 0.05 |
| Potential COVID-19 exposure | 37.8 | 85.2 | **1.12** | 83.7 | 84.0 | 0.01 | 44.9 | 74.8 | **0.64** |
| Episode overlapped with pregnancy, % | 1.7 | 2.9 | 0.09 | 1.9 | 1.8 | 0.00 | 15.7 | 9.3 | –0.20 |
| Baseline characteristics^d^ |  |  |  |  |  |  |  |  |  |
| CCI, mean score | 0.5 | 0.8 | **0.23** | 0.5 | 0.7 | **0.22** | 1.0 | 1.2 | 0.17 |
| Inpatient stay, % | 10.7 | 18.4 | **0.22** | 11.1 | 16.9 | 0.17 | 36.2 | 42.3 | 0.12 |
| ED visit, % | 34.4 | 38.7 | 0.09 | 33.0 | 38.2 | 0.11 | 51.7 | 52.6 | 0.02 |
| Outpatient^c^ visits, mean N | 7.4 | 10.8 | **0.26** | 7.5 | 10.7 | **0.23** | 16.1 | 19.8 | 0.17 |
| Prescription fills, mean N | 13.3 | 16.2 | 0.12 | 12.5 | 16.7 | 0.18 | 32.8 | 40.0 | 0.14 |

Bold red font indicates absolute (SMD) of 0.2 or more.

*CCI* Charlson Comorbidity Index, *ED* emergency department, *N/A* not applicable, *NAAT* nucleic acid amplification test, *SARS-CoV-2* severe acute respiratory syndrome coronavirus 2, *SMD* standardized mean difference

^a^ Measured at the index event

^b^ Care setting was defined using a hierarchy if multiple settings were found at the index event: inpatient, ED, outpatient, telemedicine, other

^c^ Outpatient encounters include physician office, ambulatory care, and urgent care visits

^d^ Measured across all claims during the 6 months prior to the start of the episode

Data source: OneFlorida Data Trust (source population); OneFlorida Data Trust linked Medicaid-EHR (study population). April 1–November 30, 2020.
